# Redox-mediated electrochemiluminescence enhancement for bead-based immunoassay†

**DOI:** 10.1039/d3sc06357g

**Published:** 2023-12-19

**Authors:** Alessandro Fracassa, Claudio Ignazio Santo, Emily Kerr, Sara Knežević, David J. Hayne, Paul S. Francis, Frederic Kanoufi, Neso Sojic, Francesco Paolucci, Giovanni Valenti

**Affiliations:** a Department of Chemistry Giacomo Ciamician, University of Bologna via Selmi 2 Bologna 40126 Italy g.valenti@unibo.it; b Institute for Frontier Materials, Deakin University Geelong Victoria 3220 Australia; c Univ. Bordeaux, CNRS, Bordeaux INP, Institut des Sciences Moléculaires UMR 5255 33607 Pessac France; d Deakin University, Centre for Sustainable Bioproducts, Faculty of Science, Engineering and Built Environment Geelong Victoria 3220 Australia; e Université Paris Cité, ITODYS, CNRS F-75013 Paris France; f ICMATE-CNR Corso Stati Uniti 4 35127 Padova Italy

## Abstract

Electrochemiluminescence (ECL) is a highly sensitive mode of detection utilised in commercialised bead-based immunoassays. Recently, the introduction of a freely diffusing water-soluble Ir(iii) complex was demonstrated to enhance the ECL emission of [Ru(bpy)_3_]^2+^ labels anchored to microbeads, but a comprehensive investigation of the proposed ‘redox-mediated’ mechanism was not carried out. In this work, we select three different water-soluble Ir(iii) complexes by virtue of their photophysical and electrochemical properties in comparison with those of the [Ru(bpy)_3_]^2+^ luminophore and the TPrA co-reactant. A systematic investigation of the influence of each Ir(iii) complex on the emission of the Ru(ii) labels on single beads by ECL microscopy revealed that the heterogeneous ECL can be finely tuned and either enhanced up to 107% or lowered by 75%. The variation of the [Ru(bpy)_3_]^2+^ ECL emission was correlated to the properties of each Ir(iii)-based mediator, which enabled us to decipher the mechanism of interaction and define guidelines for the future design of novel Ir(iii) complexes to further enhance the ECL emission of bead-based immunoassays. Ultimately, we showcase the potential of this technology for practical sample analysis in commercial instruments by assessing the enhancement of the collective ECL intensity from a bead-based system.

## Abstract 2

Redox mediated mechanism in electrochemiluminescence (ECL) beads-based assay: the influence of Ir(iii) redox mediators increases the ECL signal up to 107%.

## Graphical abstract

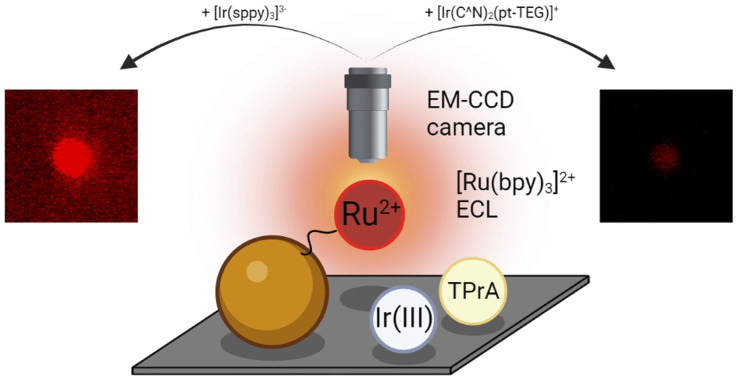

## Introduction

Electrochemiluminescence (ECL) is the process of generating light *via* strongly exergonic electron transfer reactions between reactive electrogenerated species.^1,2^ ECL offers several intrinsic advantages over conventional analytical techniques such as an excellent signal-to-noise ratio, precise spatial^3^ and temporal control, and signal generation in an aqueous environment that is essential for analysis in real matrices.^4–7^ These benefits have played a pivotal role in the establishment of ECL as a leading bioanalytical technique. To make ECL viable for clinical diagnostics, Roche Diagnostics produce fully automated analysers that exploit bead-based immunoassay technology. This system employs biotinylated antibodies and dye-functionalised antibodies that specifically recognise a given antigen. When the analyte is present, the classical sandwich assay is formed. In this way, a 1 : 1 ratio between the antigen and the labelled antibody is achieved, making the ECL proportional to the analyte concentration.^8,9^

**Fig. 1:**
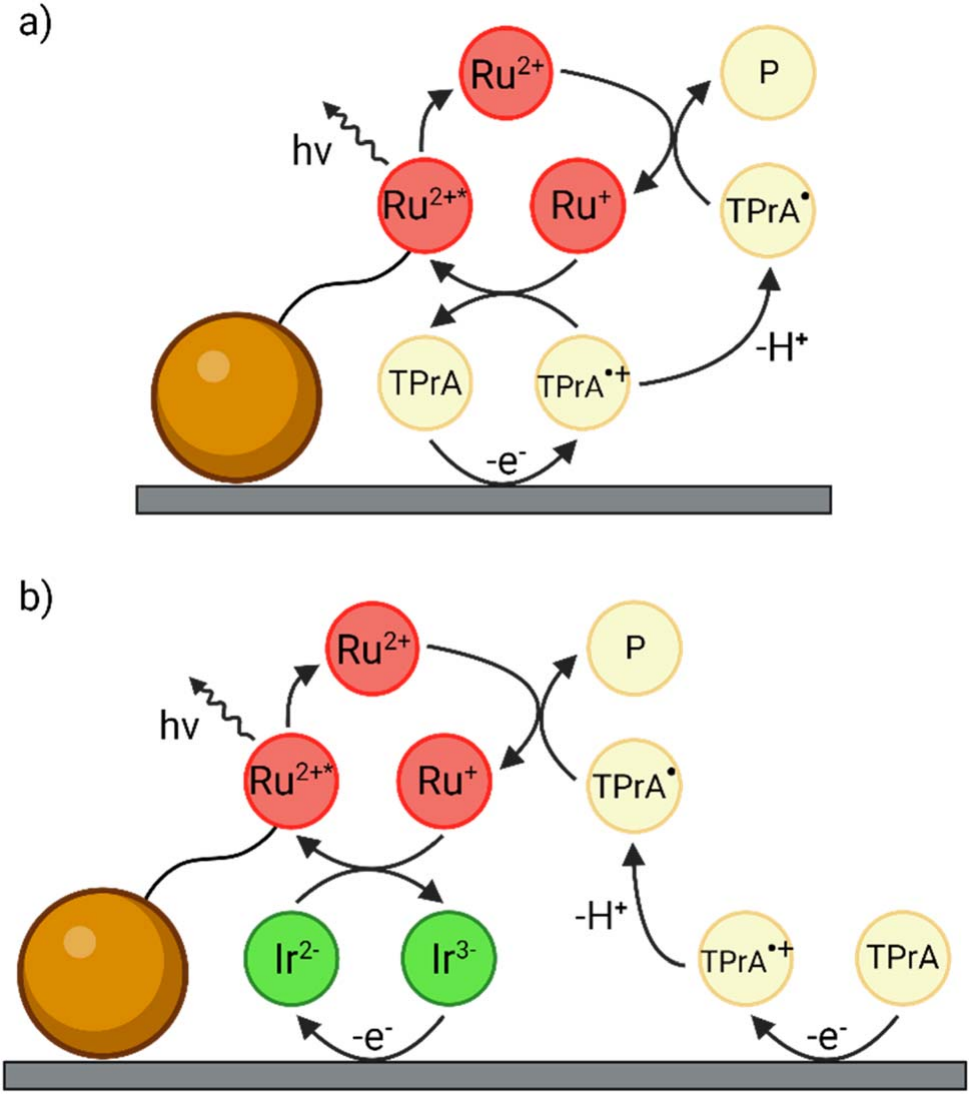
Schematics of (a) the conventional heterogeneous ECL pathway and (b) the enhanced ‘redox mediated’ pathway in a heterogeneous bead-based immunoassay. The magnetic microbead is represented by the orange sphere while the [Ru(bpy)_3_]^2+^ luminophore and the [Ir(sppy)_3_]^3−^ complex are labelled as Ru^2+^ and Ir^3−^, respectively.

Typical ECL immunoassays exploit tris(2,2′-bipyridine)ruthenium(ii) ([Ru(bpy)_3_]^2+^) and tri-*n*-propylamine (TPrA) as luminophore and co-reactant, respectively. The latter is a sacrificial molecular species that upon oxidation, undergoes an irreversible chemical step to generate strongly reducing radicals. The bead-based immunoassay follows the heterogeneous co-reactant ECL mechanism (Fig. 1a), where the Ru(ii) complexes on the beads cannot be directly oxidised because they are constrained further than the tunnelling distance of ∼1–2 nm from the electrode surface.^10^ To obtain more sensitive and reliable ECL biosensors, researchers have tried to increase the signal-to-noise ratio by employing novel luminophores with higher quantum yield compared to [Ru(bpy)_3_]^2+^,^11–14^ by introducing nanomaterials^15–22^ or by a careful molecular design of new co-reactant species.^23–26^ To date, there is a lack of straightforward methods to enhance the ECL signal of bead-based biosensors without further modifying the structure of the patented immunoassay.

In this context, Kerr *et al.* recently proposed a novel method to amplify the homogeneous ECL emission by adding a water-soluble Ir(iii) complex in solution.^27^ They investigated a solution-phase system comprising 0.3 M phosphate buffer (PB) at pH 6.8 with 0.75 μM [Ru(bpy)_3_]^2+^, 180 mM TPrA and 100 μM tris(2-(2-pyridinyl-κN)-4-sulfonatophenyl-κC)iridium(iii) ([Ir(sppy)_3_]^3−^) enabling emission from both complexes. The co-reactant ECL intensity of [Ru(bpy)_3_]^2+^ increased by 10.8-fold when applying a potential of 0.9 V *vs.* Ag/AgCl, a potential at which [Ru(bpy)_3_]^2+^ is not oxidised, and by 1.5-fold at 1.2 V, where [Ru(bpy)_3_]^2+^ is oxidised. This approach was subsequently evaluated to enhance the heterogeneous co-reactant ECL signal in the bead-based assay format.^28^ As a convenient model system, the [Ru(bpy)_3_]^2+^ luminophore was covalently bound to 12 μm beads, and 100 μM [Ir(sppy)_3_]^3−^ was introduced to the co-reactant solution. Dramatic 70.9-fold and 2.9-fold enhancements of ECL were observed at 0.9 V and 1.2 V, respectively. In these studies, the enhancement was ascribed to a ‘redox mediated’ reaction pathway (Fig. 1b), which amplifies the ECL signal of the [Ru(bpy)_3_]^2+^ labels.

**Fig. 2:**
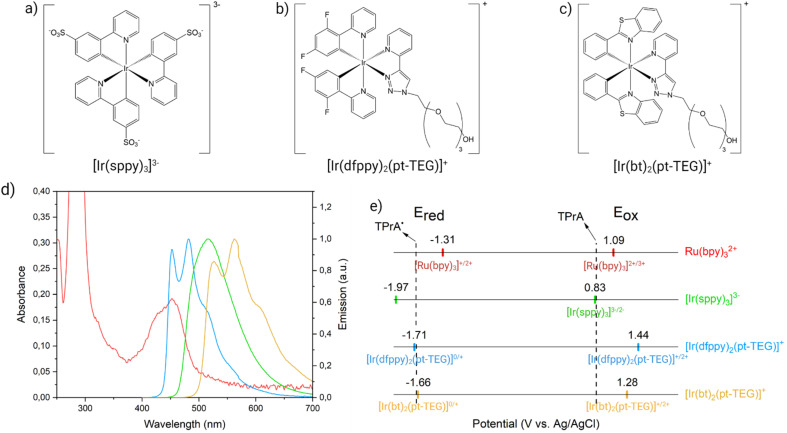
Molecular structures of water-soluble Ir(iii) complexes: (a) [Ir(sppy)_3_]^3−^, (b) [Ir(dfppy)_2_(pt-TEG)]^+^, and (c) [Ir(bt)_2_(pt-TEG)]^+^. (d) The overlap between the emission of [Ir(sppy)_3_]^3−^ (green spectrum), [Ir(dfppy)_2_(pt-TEG)]^+^ (blue spectrum), and [Ir(bt)_2_(pt-TEG)]^+^ (yellow spectrum) with the absorption of [Ru(bpy)_3_]^2+^ (red spectrum). (e) Scheme showing the reduction (*E*_red_, left) and oxidation potentials (*E*_ox_, right) of the metal complexes involved within Ru@Beads/Ir systems (same colour code as in Fig. 2d). The dashed lines show the oxidation potentials of TPrA (right) and TPrA˙ (left).

However, a thorough investigation of the interaction mechanism between [Ru(bpy)_3_]^2+^ on the beads and [Ir(sppy)_3_]^3−^ in solution has been lacking, and the pivotal features of the Ir(iii) mediator that enable its participation in the enhancement of heterogeneous ECL remain unidentified. In this work, we introduced three different water-soluble iridium complexes in a model bead-based immunoassay system, namely [Ir(sppy)_3_]^3−^ (Fig. 2a), bis(2-(2,4-difluorophenyl)pyridine)(1-(2-(2-(2-(2-hydroxyethoxy)ethoxy)ethoxy)ethyl)-1-(4-pyridil)-1,2,3-triazole)iridium(iii) ([Ir(dfppy)_2_(pt-TEG)]^+^, Fig. 2b), and bis(1,3-benzothiazole)(1-(2-(2-(2-(2-hydroxyethoxy)ethoxy)ethoxy)ethyl)-1-(4-pyridil)-1,2,3-triazole)iridium(iii) ([Ir(bt)_2_(pt-TEG)]^+^, Fig. 2c). Each Ir(iii) complex displays unique photophysical and electrochemical properties, summarised in Fig. 2, such as different emission wavelengths, redox potentials, and positive or negative charges. In particular, their emission spectra exhibit a progressively increasing degree of overlap with the absorption spectrum of [Ru(bpy)_3_]^2+^ (Fig. 2d and Table S1†). At the same time, each complex presents different electrochemical properties that modulate to different extent the homogeneous electron transfer with both the TPrA co-reactant and the α-aminoalkyl radical TPrA˙ (Fig. 2e and Table S1†).

Herein, we utilise ECL microscopy (see Fig. S1†)^29–32^ to analyse the emission of Ru(ii) labels attached to magnetic beads upon interaction with each Ir(iii) mediator. The results were compared to the ECL signal of single beads generated at the same potential in a co-reactant solution without any Ir(iii) complex. By systematically varying both the Ir(iii) mediator and the experimental conditions (*i.e.*, Ir(iii) mediator concentration, TPrA concentration, working electrode potential), we were able to achieve an ECL enhancement up to 107%, or quenching by up to 75%. Notably, while observing a comparable ECL enhancement to that reported in our previous work under similar conditions, we also describe circumstances where the ECL signal is partially switched off. By correlating the properties of each freely diffusing Ir(iii) complex to their influence on the ECL emission of a model bead-based immunoassay, we present for the first time a comprehensive study of this novel reaction mechanism. Our findings enable the development of guidelines for the future design of novel redox mediators that maximise the effectiveness of such mechanism. Finally, we transitioned from the ECL microscopy to a collective beads experimental setup in which the entirety of the ECL generated by the beads is collected by a photomultiplier tube (PMT). The introduction of 50 μM [Ir(sppy)_3_]^3−^ into the co-reactant solution resulted in a substantial 22.3% enhancement of the ECL signal from the [Ru(bpy)_3_]^2+^ labels on the beads, demonstrating the potential application of this technology for real sample analysis in commercial instruments.

## Experimental section

### Chemical

Tri-*n*-propylamine (TPrA, MW = 143.27 g mol^−1^, ≥98% v/v), sodium phosphate monobasic dihydrate (NaH_2_PO_4_·2H_2_O, MW = 156.01 g mol^−1^, ≥99%), sodium phosphate dibasic (Na_2_HPO_4_, MW = 141.96 g mol^−1^, ≥99.5%) and phosphoric acid (H_3_PO_4_, MW = 98.00 g mol^−1^, ≥85%) were purchased from Sigma-Aldrich. 2.8 μm polystyrene magnetic beads covalently linked to [Ru(bpy)_3_]^2+^ were purchased from Roche Diagnostics. Na_3_[Ir(sppy)_3_] was purchased from Luminescence Technology Corp. while [Ir(dfppy)_2_(pt-TEG)]Cl and [Ir(bt)_2_(pt-TEG)]Cl were synthesised as reported in literature.^13^

### Photophysical characterisation

The absorption spectrum of [Ru(bpy)_3_]^2+^ was recorded as reported in literature,^13^ using a 10 μM solution of the complex in ultra-pure (Milli-Q) water at room temperature. The acquired spectrum was normalised for comparative purposes. The emission spectra of [Ir(sppy)_3_]^3−^ (Fig. 2d, green line), [Ir(dfppy)_2_(pt-TEG)]^+^ (Fig. 2d, blue line) and [Ir(bt)_2_(pt-TEG)]^+^ (Fig. 2d, yellow line) were collected as reported in literature.^13,27^ They were recorded by exciting the samples at 340 nm using 10 μM solutions in ultra-pure water at room temperature.

The resulting spectra were corrected for the change in instrument sensitivity over the wavelength range and then normalised.

### Electrochemical characterisation

The electrochemical characterisation of [Ir(dfppy)_2_(pt-TEG)]^+^, [Ir(sppy)_3_]^3−^ and [Ir(bt)_2_(pt-TEG)]^+^ was performed as reported in literature.^15,26,27,32,33^ The *E*_ox_ for [Ir(sppy)_3_]^3−^ was determined by computing *E*_1/2_ of the [Ir(sppy)_3_]^3−/2−^ couple from a cyclic voltammetry experiment carried out in PB 0.1 M with 1 mM [Ir(sppy)_3_]^3−^ (scan rate, 100 mV s^−1^).^28^ The *E*_ox_ for the [Ir(C^N)_2_(pt-TEG)]^+^ complexes were retrieved from a 0.25 mM coordination compound solution in 0.1 M PB by square wave voltammetry (step, 5 mV; amplitude, 0.02; frequency, 25 Hz).^13^ The *E*_ox_ for [Fe(bpy)_3_]^2+^ was determined by computing *E*_1/2_ of the [Fe(bpy)_3_]^2+/3+^ couple from a cyclic voltammetry experiment carried out in KCl 0.5 M with 1 mM [Fe(bpy)_3_]^2+^ (scan rate, 100 mV s^−1^).^32^ The *E*_red_ values were determined by cyclic voltammetry in DMF with 0.1 M TBAPF_6_ electrolyte for [Ir(sppy)_3_]^3−^ and 0.1 M TBABF_4_ for [Fe(bpy)_3_]^2+^,^33^ and in ACN with 0.1 M TBAPF_6_ electrolyte for the [Ir(C^N)_2_(pt-TEG)]^+^ complexes (scan rate, 100 mV s^−1^).

### Beads preparation

Prior to use, magnetic beads covalently functionalised with [Ru(bpy)_3_]^2+^ were washed in a phosphate buffer (PB) solution and sonicated for 15 minutes.

### Electrochemiluminescence

In the microscopy setup, the ECL and optical images were captured following the injection of a suspension of [Ru(bpy)_3_]^2+^ covalently functionalised beads in the electrochemical cell where the microspheres were collected on the working electrode surface using a magnet placed underneath. The ECL and optical imaging was performed using solutions of 0.3 M PB, variable TPrA concentrations (pH 6.8) and a redox mediator, in a homemade PTFE electrochemical cell comprising a Pt working (0.16 cm^2^), Pt counter, and Ag/AgCl (3 M KCl) reference electrodes. The different solutions were inserted in the electrochemical cell with a pressure-driven flow controller (OB1 Mk3, Elveflow) equipped with a flux sensor (Flow-04D working range from 0 to 1000 μL min^−1^) and exchanged, when necessary, with a 10-way bidirectional valve (MUX distributor). For microscopic imaging, an epifluorescence microscope from Nikon (Chiyoda, Tokyo, Japan) equipped with an ultrasensitive EMCCD camera (EM-CCD 9100-13 from Hamamatsu, Japan) was used with a resolution of 512 × 512 pixel and a size of 16 × 16 μm^2^. The microscope was enclosed in a homemade dark box to avoid interferences from external light. It was also equipped with a motorised microscope stage (Corvus, Märzhauser, Wetzlar, Germany) for sample positioning and with long-distance objective from Nikon (magnification 100×/numerical aperture 0.80/DL 4.5). Additionally, the integrated system included a SP-300 potentiostat (BioLogic Science Instrument, France) triggered with the camera (see Fig. S1†). CV-ECL plots were collected by scanning the working electrode potential at 100 mV s^−1^ from open circuit potential (OCP) up to 2 V (*vs.* Ag/AgCl 3 M KCl), back to 0 V (*vs.* Ag/AgCl 3 M KCl) and, eventually, terminating the cycle at OCP. The beads emission during CV-ECL measurements was acquired every 200 ms to follow the temporal evolution of the signal. The integration time of the EM-CCD camera was set to 200 ms. ECL images were recorded by applying a double chronoamperometric pulse: OCP for 2 s and a suitable potential (*vs.* Ag/AgCl 3 M KCl) for the next 8 s. The total integration time of the EM-CCD camera was set to 10 s. Unless otherwise stated, gain and sensitivity parameters of the EM-CCD camera were set to 1 and 255, respectively.

In the collective beads configuration, 5.5 μL of a 0.72 mg mL^−1^ suspension of [Ru(bpy)_3_]^2+^ covalently functionalised beads were deposited on the working electrode surface, where they stick due to a magnet placed beneath. ECL measurements were carried out using 3 mL of solutions comprised of 0.3 M PB, 180 mM TPrA (pH 6.8) and a redox mediator (where required), in a PTFE homemade electrochemical cell comprising a Pt working (0.3 cm^2^), Pt counter, and Ag/AgCl (3 M KCl) reference electrodes. The ECL signal was collected by a photomultiplier tube (PMT) positioned on top of the cell, whose voltage was set to 750 V. The recorded emission was amplified to a 000.0 μA level using a Keithley Model 6485 Picoammeter (Keithley Instruments Inc., Ohio, United States). Between the cell and the PMT we placed a longpass filter with a cut-on wavelength of 606 nm (Newport Corporation, Irvine, California, USA) to maximise the isolation of the ECL emission of [Ru(bpy)_3_]^2+^ labels from the Ir(iii) complexes. The system was enclosed in a homemade dark box to avoid interferences from external light. The ECL emission is triggered by anodic potential sweep during cyclic voltammetry controlled by a SP-300 potentiostat.

## Results and discussion

Generally, ECL sandwich immunoassay involves the capture of the antibody–antigen complex; therefore, as a proof of concept, we decided to employ magnetic beads decorated with Ru(ii) labels. These covalently functionalised 2.8 μm beads (Ru@Beads) mimic the activity of the bead-based ECL immunoassay and generate an improved signal-to-noise ratio due to the higher surface concentration of Ru(ii) labels. In this case, this feature is crucial to emphasise the beads signal over the background luminescence generated by homogeneous ECL from the Ir(iii) complex.

We characterised the reference [Ru(bpy)_3_]^2+^-labelled bead system *via* cyclic voltammetry-ECL (CV-ECL) measurement (see Fig. S2 and ESI Video 1†) on a Pt working electrode (WE) in 0.3 M PB at pH 6.8 with 180 mM TPrA as a sacrificial co-reactant. Starting at 0.83 V, TPrA is oxidised at the electrode yielding TPrA˙^+^. This aminium radical cation is unstable (*t*_1/2_ ∼200 μs) and rapidly deprotonates to form the strongly reducing α-aminoalkyl radical TPrA˙ which converts [Ru(bpy)_3_]^2+^ to [Ru(bpy)_3_]^+^. The reduced luminophore is then oxidised by TPrA˙^+^ yielding the emitting [Ru(bpy)_3_]^2+^* (Fig. 1a).^10,35,36^ The short half-life of TPrA˙^+^ represents an intrinsic limitation to the signal intensity since the radical cations diffuse only within a limited region from the electrode surface, confining the ECL emitting layer to ∼3 μm (*i.e.*, only the luminophores located in the ECL reactive layer, where both TPrA˙^+^ and TPrA˙ coexist, are involved in the ECL process; see Scheme S1†).^37–44^

**Fig. 3:**
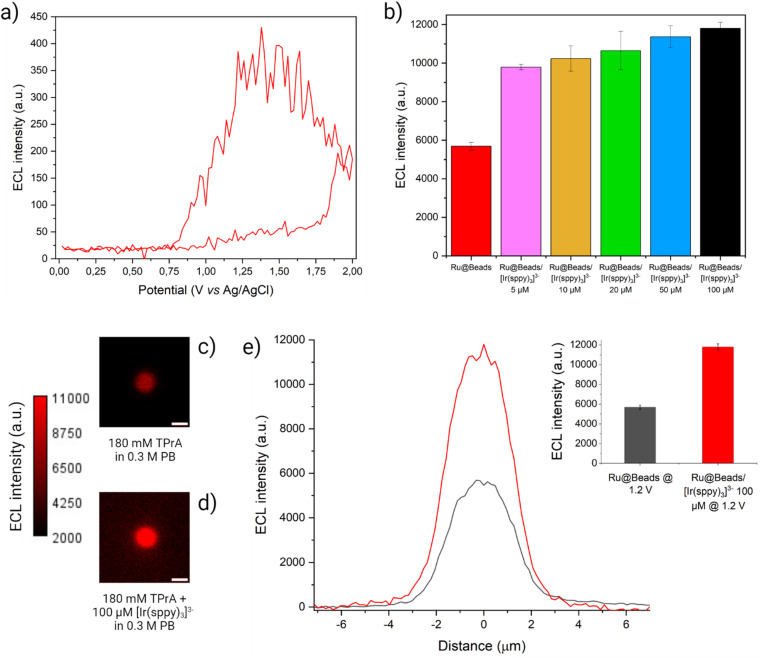
(a) CV-ECL measurement performed on Ru@Beads/[Ir(sppy)_3_]^3−^ in a 0.3 M PB solution at pH 6.8 with 180 mM TPrA and 100 μM [Ir(sppy)_3_]^3−^. The working electrode potential was scanned at 100 mV s^−1^ from OCP up to 2 V (*vs.* Ag/AgCl), back to 0 V (*vs.* Ag/AgCl) and, eventually, terminating the cycle at OCP. The beads ECL emission (red line) was acquired each 200 ms and, for each frame, the maximum value of the beads ECL profile is plotted *versus* the applied potential. The background signal is eliminated by subtracting, for each frame, the average ECL intensity value of the background retrieved over a 50 × 50 pixel square centred in a region of the image where no beads are present. (b) Comparison between ECL intensities of Ru@Beads (red bar) and of Ru@Beads/[Ir(sppy)_3_]^3−^ in a 0.3 M PB solution at pH 6.8 with 180 mM TPrA and different [Ir(sppy)_3_]^3−^ concentrations: 5 μM (pink bar), 10 μM (yellow bar), 20 μM (green bar), 50 μM (blue bar) and 100 μM (black bar). The ECL intensities were obtained from ECL images captured using an EM-CCD camera during a two-step chronoamperometry measurement: 2 s at OCP and 8 s at 1.2 V *vs.* Ag/AgCl. Magnification, 100×; objective numerical aperture, 0.8; gain, 1; sensitivity, 255. Data are averaged over a minimum of six beads (*n* ≥ 6). Each bar represents the maximum value of the respective ECL profile, and the error bars show the standard errors. (c and d) ECL images of a single 2.8 μm bead covalently labelled with [Ru(bpy)_3_]^2+^ in a 0.3 M PB solution at pH 6.8 with 180 mM TPrA (c) without (Ru@Beads) and (d) with 100 μM [Ir(sppy)_3_]^3−^ (Ru@Beads/[Ir(sppy)_3_]^3−^). The background signal in (d) is generated by [Ir(sppy)_3_]^3−^* following the conventional homogeneous ECL pathways. The images were obtained with an EM-CCD camera by recording the ECL signal for 10 s during a two-step chronoamperometry measurement: 2 s at open circuit potential (OCP) and 8 s at 1.2 V *vs.* Ag/AgCl. Magnification, 100×; objective numerical aperture, 0.8; gain, 1; sensitivity, 255; contrast intensity scale: 2000 to 11 000; scale bar: 3 μm. (e) Comparison between the single-bead ECL intensity profiles of Ru@Beads (grey line) and Ru@Beads/[Ir(sppy)_3_]^3−^ (red line). Inset: histogram of the comparison between the respective averaged maximum values of ECL intensity where the error bars show the standard error. Data are averaged over a minimum of six beads (*n* ≥ 6).

We repeated CV-ECL measurements to test the effects of the addition in solution of each freely diffusing Ir(iii) complex on the ECL signals of the Ru@Beads: hereafter, these systems will be denoted as Ru@Beads/[Ir(sppy)_3_]^3−^, Ru@Beads/[Ir(dfppy)_2_(pt-TEG)]^+^, and Ru@Beads/[Ir(bt)_2_(pt-TEG)]^+^ indicating the addition of the corresponding Ir(iii) complex (see Fig. 3a, S3, S4 and ESI Video 2†). Ultimately, we performed integrated ECL measurements where the [Ru(bpy)_3_]^2+^ contribution to the overall emission is quantified by extracting the background-subtracted ECL profiles (as reported in ECL images elaboration, Fig. S5–S9†) from the corresponding integrated images. In other words, the resulting ECL intensity profiles are solely attributed to the emission of the [Ru(bpy)_3_]^2+^ labels. The background-subtracted profiles were then compared to those generated by Ru@Beads at the same potential in the absence of the Ir(iii) mediator.

The ECL intensity of Ru@Beads/[Ir(sppy)_3_]^3−^ collected at 1.2 V showed a 72% enhancement when adding 5 μM [Ir(sppy)_3_]^3−^ in solution, as compared to Ru@Beads. The emission increased up to 107% at 100 μM, thus proving the ability of [Ir(sppy)_3_]^3−^ to enhance the ECL signal (see Fig. 3 and S10†). However, the enhancement provided by the addition of [Ir(sppy)_3_]^3−^ is progressively less pronounced at increasing concentration, eventually leading to a plateau (see Fig. S11 and S12†). This shows that increasing the concentration of [Ir(sppy)_3_]^3−^ does not necessarily correspond to a proportional enhancement of Ru@Beads emission. Intrigued by this behaviour, we explored the ECL signal gain of Ru@Beads/[Ir(sppy)_3_]^3−^ compared to Ru@Beads at significantly greater [Ir(sppy)_3_]^3−^/TPrA concentration ratios. Both systems had their TPrA concentration reduced from 180 mM to 25 mM, while the concentration of [Ir(sppy)_3_]^3−^ in Ru@Beads/[Ir(sppy)_3_]^3−^ was maintained at 100 μM. Under these conditions, the variation in emission intensity revealed an unexpected trend as the signal gain decreased with increasing [Ir(sppy)_3_]^3−^/TPrA ratio, eventually resulting in a −17% quenching of the ECL at 25 mM of TPrA (see Fig. S13–S19†). It is likely that beyond a certain [Ir(sppy)_3_]^3−^/TPrA threshold, the competing reaction between the oxidised Ir(iv) and TPrA˙ (see Reaction (S2.5)†) prevails, leading to an increase of [Ir(sppy)_3_]^3−^ background emission rather than to the enhancement of the Ru@Beads ECL signal. Therefore, the optimal concentration ratio for maximising the enhancement could be in the order of ∼5 × 10^−4^.

**Fig. 4:**
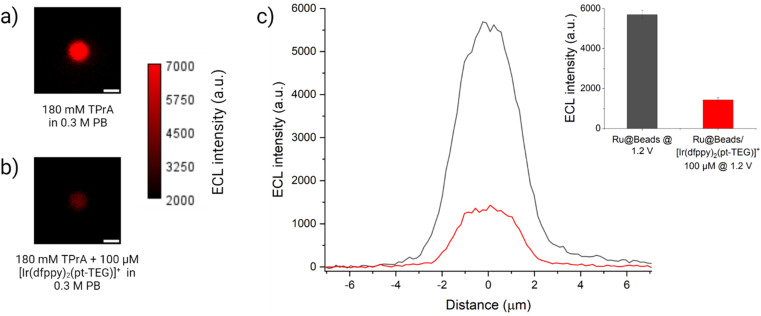
ECL images of a 2.8 μm single-bead covalently labelled with [Ru(bpy)_3_]^2+^ in a 0.3 M PB with 180 mM TPrA (Ru@Beads) (a) without and (b) with 100 μM of [Ir(dfppy)_2_(pt-TEG)]^+^. The background signal in (b) is generated by [Ir(dfppy)_2_(pt-TEG)]^+^* following the conventional homogeneous ECL pathways. The images were obtained with an EM-CCD camera by recording the ECL signal for 10 s during a two-step chronoamperometry measurement: 2 s at open circuit potential (OCP) and 8 s at 1.2 V *vs.* Ag/AgCl. Magnification, 100×; objective numerical aperture, 0.8; gain, 1; sensitivity, 255; contrast scale: 2000–7000. Scale bar: 3 μm. (c) Comparison between the single-bead ECL intensity profiles of Ru@Beads (grey line) and Ru@Beads/[Ir(dfppy)_2_(pt-TEG)]^+^ (red line). Inset: histogram of the comparison between the respective averaged maximum values of ECL intensity where the error bars show the standard error. Data are averaged over a minimum of six beads (*n* ≥ 6).

Similarly, integrated ECL measurements were carried out on Ru@Beads/[Ir(dfppy)_2_(pt-TEG)]^+^ using 100 μM of the Ir(iii) complex. In contrast to [Ir(sppy)_3_]^3−^, which exhibits nearly identical oxidation potential to TPrA, [Ir(dfppy)_2_(pt-TEG)]^+^ necessitates a significantly more anodic potential (*E*_ox_ = 1.44 V) to be oxidised. By modulating the working electrode potential, one can effectively control the presence of oxidised Ir(iv) species and investigate the ECL response in scenarios where only Ir(iii) species are present in solution or where both Ir(iii) and Ir(iv) species coexist. Essentially, the applied potential can work as a switch, enabling the manipulation of chemical conditions to tune the reaction mechanism. At first, a potential of 1.5 V was imposed to achieve the oxidation of [Ir(dfppy)_2_(pt-TEG)]^+^. Quite surprisingly, instead of an enhancement, we observed a 24% drop in ECL intensity compared to Ru@Beads (see Fig. S20†). Moreover, at 1.2 V, where only the co-reactant is oxidised, the ECL intensity suffers an even more pronounced attenuation (−75%) compared to the Ru@Beads reference (Fig. 4). Thus, contrary to [Ir(sppy)_3_]^3−^, this complex decreases the [Ru(bpy)_3_]^2+^-emitted ECL either in presence or in absence of the oxidised Ir(iv) species.

Finally, the Ru@Beads/[Ir(bt)_2_(pt-TEG)]^+^ system was studied at two distinct potentials as well: 1.3 V, where both the co-reactant and [Ir(bt)_2_(pt-TEG)]^+^ (*E*_ox_ = 1.28 V) are oxidised; and 1.2 V, at which TPrA is exclusively oxidised. At 1.3 V, the ECL signal gradually decreased when increasing the [Ir(bt)_2_(pt-TEG)]^+^ concentration, which was the opposite trend to that observed for Ru@Beads/[Ir(sppy)_3_]^3^. Compared to Ru@Beads, the ECL intensity of Ru@Beads/[Ir(bt)_2_(pt-TEG)]^+^ decreased from −50% at 10 μM Ir(iii) complex concentration to −65% at 100 μM (see Fig. S21–S23†). Similarly, by introducing 100 μM [Ir(bt)_2_(pt-TEG)]^+^ and applying 1.2 V, the ECL intensity experiences a quenching effect of −71% (see Fig. S24 and S25†).

A rationale for the contrasting behaviour of both [Ir(dfppy)_2_(pt-TEG)]^+^ and [Ir(bt)_2_(pt-TEG)]^+^ with respect to [Ir(sppy)_3_]^3−^ can be found in the thermodynamics of the various Ru@Beads/TPrA/Ir(iii) systems (see Schemes S2–S4†). The three Ir(iii) complexes differ from one another in their reduction and oxidation potentials (Fig. 2e), which results in mediators with different reducing and oxidising strengths during the ECL process. At 1.2 V, [Ir(sppy)_3_]^3−^ is the only Ir(iii) complex that will be oxidised. Although capable of scavenging TPrA˙ *via* homogeneous oxidation (see Reaction (S2.5)†), this Ir(iv) species also opens two alternative paths for [Ru(bpy)_3_]^2+^ ECL emission: the homogeneous oxidation of TPrA (*i.e.*, catalytic route, see Reaction (S2.3)†) and the mediated oxidation of the [Ru(bpy)_3_]^+^ species (see Fig. 1b and Reaction (S2.8)†). These reactions provide a plausible explanation for the observed ECL enhancement. The feasibility of the catalytic route and its impact on the ECL emission of single beads are demonstrated in CV-ECL by the anticipation of 65 mV of the ECL onset potential when introducing 100 μM of [Ir(sppy)_3_]^3−^ in the co-reactant solution that yield a signal enhancement of 199% at 0.85 V (see Fig. S2,† and 2a). On the other hand, evidence for the redox mediated pathway is seen in the mixed annihilation ECL reaction of [Ru(bpy)_3_]^2+^ and [Ir(sppy)_3_]^3−^ in 4 : 1 acetonitrile/water, when −1.4 V and 0.96 V are alternatingly applied to selectively reduce [Ru(bpy)_3_]^2+^ and oxidise [Ir(sppy)_3_]^3−^.^27^ Ir(iv) species can be invoked at more oxidising electrode potentials for both [Ir(dfppy)_2_(pt-TEG)]^+^ and [Ir(bt)_2_(pt-TEG)]^+^. These could be anticipated to generate [Ru(bpy)_3_]^2+^* *via* analogous reactions, although they are much stronger oxidants than [Ir(sppy)_3_]^2−^ and may also catalyse the oxygen evolution reaction.

The observed quenching behaviour at 1.2 V for both [Ir(dfppy)_2_(pt-TEG)]^+^ and [Ir(bt)_2_(pt-TEG)]^+^, however, can be ascribed to the potentials at which they are reduced. For both species, this occurs at almost the same potential at which TPrA˙ is oxidised, whereas [Ir(sppy)_3_]^3−^ is more difficult to reduce by *ca.* 300 mV. The only thermodynamically feasible reaction that could explain the quenching behaviour is the homogeneous reduction of the [Ir(C^N)_2_(pt-TEG)]^+^ complexes by TPrA˙ (see Reactions (S3.5) and (S4.5)†). The similar quenching magnitude between Ru@Beads/[Ir(dfppy)_2_(pt-TEG)]^+^ and Ru@Beads/[Ir(bt)_2_(pt-TEG)]^+^ at 1.2 V implies that both [Ir(C^N)_2_(pt-TEG)]^+^ complexes share the same mechanism and act as scavengers toward the reducing TPrA˙ radical, which is essential for both the unenhanced and enhanced ECL pathways (Fig. 1a and b, respectively). In this context, the similar degree of quenching expressed by Ru@Beads/[Ir(bt)_2_(pt-TEG)]^+^ at both 1.2 and 1.3 V suggests that the strongly oxidising Ir(iv) complex plays only a marginal role in buffering the [Ru(bpy)_3_]^2+^ emission quenching *via* electron transfer. To further support the importance of the strength of the electrogenerated oxidiser, we recorded the ECL emission of Ru@Beads in a solution of 180 mM TPrA with 100 μM of the non-emitting [Fe(bpy)_3_]^2+^ (*E*_ox_ = 0.85 V,^33^*E*_red_ = −1.22 V,^34^ Ru@Beads/[Fe(bpy)_3_]^2+^) at 1.2 V. The presence of [Fe(bpy)_3_]^2+^ quenches the ECL emission of the standard Ru@Beads by 46% due to the scavenging effect of the [Fe(bpy)_3_]^2+^ complex towards TPrA˙ (see Fig. S26 and S27†). However, the quenching magnitude is less pronounced compared to both Ru@Beads/[Ir(C^N)_2_(pt-TEG)]^+^ at 1.2 V. This effect is associated, as for [Ir(sppy)_3_]^3−^, to the thermodynamic stability of the oxidised Fe(iii) redox state (counterpart of Ir(iv) for [Ir(sppy)_3_]^3−^) that is much less reactive than the oxidised [Ir(C^N)_2_(pt-TEG)]^2+^ species and thus can effectively participate in the mediated oxidation of the [Ru(bpy)_3_]^+^ labels. In brief, both [Ir(C^N)_2_(pt-TEG)]^+^ complexes work as radicals scavengers while [Ir(sppy)_3_]^3−^ is the only Ir(iii) complex amplifying the ECL signal of the labelled beads.

All of the above supports electron transfer as the interaction mechanism leading either to heterogeneous ECL enhancement or quenching. We could actually exclude energy transfer as a major contributor to such an effect because of the poor overlap between the emission spectrum of [Ir(sppy)_3_]^3−^ with the absorption spectrum of [Ru(bpy)_3_]^2+^.

The opposite enhancement and quenching phenomena result from redox mediated mechanisms involving either the oxidised or reduced forms of the Ir(iii) species, depending on their standard oxidation and reduction potentials relatively to those of the TPrA. The ECL of the Ru@Beads is kinetically controlled by the availability of short-lived TPrA˙^+^ and TPrA˙ species to react with the Ru(ii)-anchored labels, and the alternative oxidants or reductants provided by the introduction of Ir(iii) complexes thus results in considerable variation in the ECL emission intensity. Given the large excess of co-reactant in solution and the similarity between their oxidation potentials,^45,46^ the oxidised [Ir(sppy)_3_]^2−^ complex (as with the oxidised [Fe(bpy)_3_]^3+^ complex) homogeneously oxidise the TPrA to TPrA˙^+^. This homogeneous generation of TPrA˙^+^ offers a kinetic advantage, especially at low overpotentials, compared to the sluggish electron transfer associated with the heterogeneous TPrA oxidation on a Pt electrode.^47^ This ultimately results in an increased production of TPrA radical. The catalytic route, altogether with the ability of the Ir(iv) species to oxidise the reduced [Ru(bpy)_3_]^+^ species, results in the large ECL enhancement in the potential region where the TPrA oxidation is under kinetic control. On the other hand, at 1.2 V (*i.e.*, diffusion-controlled regime for TPrA oxidation), most of the current can be attributed to the direct electro-oxidation of TPrA and, in turn, most of the ECL signal arises from reaction with electrogenerated TPrA˙^+^ (Fig. S11†). Therefore, we hypothesise that the homogeneous oxidation of the co-reactant may provide a significant contribution at less anodic potentials (*i.e.*, 0.9 V) where the oxidation of the TPrA at the electrode is notably sluggish, but at 1.2 V, the redox mediated pathway shown in Fig. 1b is the most effective. In this context, the negative charge on the [Ir(sppy)_3_]^3−^ complex could promote its approach to the ECL label due to coulombic attraction, increasing the rate of interactions. In order to elucidate such ‘redox mediated’ ECL, we propose a simplified model (see ESI Section 13†) combining a steady-state formalism to describe the surface concentration of the Ru-based species and a COMSOL simulation of the electrochemistry of TPrA and Ir species in solution. The ECL signal enhancement provided by the introduction of 100 μM of [Ir(sppy)_3_]^3−^ upon application of 1.2 V can be reproduced (see modelled ECL profile in Fig. S28a†) without any increase in the emitting layer thickness by considering both the redox mediated formation of the emitter (Fig. 1b) and the redox mediated oxidation of the co-reactant:[Ir(sppy)_3_]^2−^ + [Ru(bpy)_3_]^+^ → [Ir(sppy)_3_]^3−^ + [Ru(bpy)_3_]^2+^*[Ir(sppy)_3_]^2−^ + TPrA → [Ir(sppy)_3_]^3−^ + TPrA˙^+^

For the other two complexes (Fig. S28b†), the quenching of the ECL signal is consistent with TPrA˙ scavenging:[Ir(C^N)_2_(pt-TEG)]^+^ + TPrA˙ → [Ir(C^N)_2_(pt-TEG)]^0^ + Pwhere P is the TPrA oxidation product. It is noteworthy that these complexes also allow the confinement of the TPrA˙ radical in the vicinity of the electrode surface, preventing radical chemistry path (or reactive radical species formation) in the solution bulk. This may be adventitious for further use of TPrA as co-reactant for ECL imaging of real biological systems. The extent of the ECL quenching can be modulated by considering the contribution of the inter-species electron transfer (see Reactions (S3.11) and (S4.11),† see modelled ECL profile in Fig. S28b†), where the faster this step, the weaker the quenching:[Ir(C^N)_2_(pt-TEG)]^0^ + [Ru(bpy)_3_]^2+^ → [Ir(C^N)_2_(pt-TEG)]^+^ + [Ru(bpy)_3_]^+^In both situations, the simplified computed ECL model could indeed reproduce the observed trends at 1.2 V. Finally, the involvement of all redox states from a redox mediator, as for the [Fe(bpy)_3_]^2+^ case, could also be reproduced (see modelled ECL profile in Fig. S28c†).

**Fig. 5:**
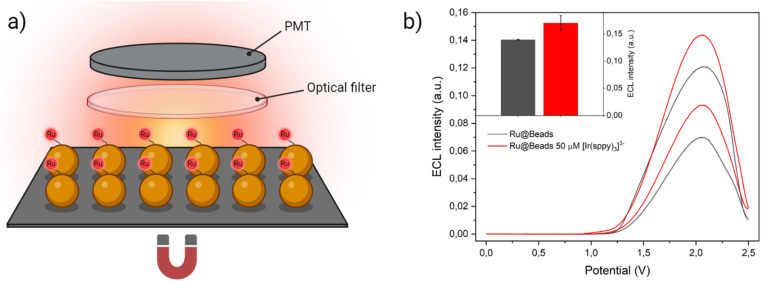
(a) Experimental setup employed to record the ECL signal in collective beads configuration. The beads were deposited on the surface of a Pt working electrode where they remain because of a magnet placed underneath. To discern the emission of [Ru(bpy)_3_]^2+^ labels from the homogeneous ECL of [Ir(sppy)_3_]^3−^, the ECL generated during the anodic potential sweep first passed through an optical filter that cuts off the all the light below 606 nm and, eventually, strikes the PMT that capture all the light without spatially resolving the signal. Yet, a small but non-negligible background signal due to [Ir(sppy)_3_]^3−^ homogeneous ECL could still be detected, thus the ECL intensity generated by Beads/[Ir(sppy)_3_]^3−^ (*i.e.*, ECL signal of the same amount of non-labelled streptavidin-coated beads in the co-reactant solution with 50 μM of [Ir(sppy)_3_]^3−^) was subtracted to Ru@Beads/[Ir(sppy)_3_]^3−^ during data processing. (b) CV-ECL measurement performed on Ru@Beads (grey line) and of Ru@Beads/[Ir(sppy)_3_]^3−^ at 50 μM concentration of [Ir(sppy)_3_]^3−^ (red line), both in a 0.3 M PB solution at pH 6.8 with 180 mM TPrA. The working electrode potential was scanned at 100 mV s^−1^ from OCP up to 2.5 V (*vs.* Ag/AgCl), back to 0 V (*vs.* Ag/AgCl) and, eventually, terminating the cycle at OCP. The inset represents a comparison between ECL intensities of Ru@Beads (grey bar) and of Ru@Beads/[Ir(sppy)_3_]^3−^ at 50 μM concentration of [Ir(sppy)_3_]^3−^ (red bar) in a 0.3 M PB solution at pH 6.8 with 180 mM TPrA. Each bar represents the ECL intensity obtained by integrating the whole CV-ECL cycle and the error bars show the standard errors. Data are averaged over two different measurements.

Leveraging our deepened understanding of the impact of Ir(iii) redox mediators on the ECL emission of a bead model system, we have extended our investigation to showcase a tangible application of the addition of [Ir(sppy)_3_]^3−^ in the co-reactant solution by emulating the experimental conditions commonly employed in clinical bioanalysis. The transition from the original microscopy configuration to a collective configuration, utilising a photomultiplier tube (PMT) instead of an EM-CCD camera, enabled the comprehensive capture of emitted light from all the beads immobilised on the working electrode surface (Fig. 5a). Given the absence of spatial resolution, distinguishing the [Ru(bpy)_3_]^2+^ labels emission from the homogeneous ECL of [Ir(sppy)_3_]^3−^ would, in principle, seem challenging. To address this, we introduced a longpass optical filter (*λ*_cut-on_ = 606 nm) to minimise the background emission from the Ir(iii) species while still capturing the peak of the [Ru(bpy)_3_]^2+^ emission (see Fig. S29†). Any residual background signal attributed to [Ir(sppy)_3_]^3−^ homogeneous ECL was subsequently subtracted during the data processing stage (see Fig. S30†).

Upon integrating the ECL signal generated during the CV-ECL cycle, we determined that the addition of 50 μM of [Ir(sppy)_3_]^3−^ yields a 22.3% enhancement in beads emission (Fig. 5b). However, it is noteworthy that the signal gain observed in the two experimental setups, under the same chemical conditions, displays marked difference. The enhancement achieved in collective beads configuration is much smaller, and this divergence can be ascribed to the spatial arrangement of the beads. While in microscopy experiments, the beads were injected into the cell, ensuring a uniform spatial distribution across the electrode surface, in the collective setup they were directly deposited on top of the working electrode where they tend to cluster together. This clustering hinders the ideal diffusion of TPrA radicals and electro-oxidised [Ir(sppy)_3_]^2−^ to the core, thereby resulting in a less pronounced ECL enhancement.

## Conclusions

We have demonstrated that the ECL reactions between [Ru(bpy)_3_]^2+^ and three Ir(iii) complexes involve an electron transfer mechanism in which the quenching or enhancing nature of the redox mediators is dependent on their redox potentials. More facile reduction yields scavenging of the critical radical species derived from the co-reactant and therefore quenching of the ECL of [Ru(bpy)_3_]^2+^. On the other hand, the production of the oxidised Ir(iv) species may boost the ECL, particularly when the reduction route is not thermodynamically feasible (such as for [Ir(sppy)_3_]^3−^). These findings provide a new framework to design mediators to further enhance the ECL signal-to-noise ratio in bead-based assays.

Ultimately, the proposed shift in experimental design provides a concrete example of the relevance of this strategy in a setting that mirrors industrial bioanalysis practices, bridging the gap between fundamental understanding and real-sensor application.

## Data availability

Experimental data and ECL images are available at AMS Acta at https://amsacta.unibo.it/id/eprint/7289.

## Author contributions

E. K. and D. J. H. performed the synthesis, characterisations of the complexes and analysed the data. A. F. and C. I. S. performed the ECL experiments. S. K., G. V. and F. K. performed the simulation of concentration profile. P. S. F., F. K., N. S., F. P. and G. V. directed the study and designed the research. All authors prepared the manuscript and approved the final version.

## Conflicts of interest

The authors declare no competing financial interest.

## Supplementary Material

SC-015-D3SC06357G-s001

SC-015-D3SC06357G-s002

SC-015-D3SC06357G-s003
