# Supplementary material for: Redox-mediated electrochemiluminescence enhancement for bead-based immunoassay
Source: Chem Sci. 2023 Dec 19;15(3):1150–8. doi: 10.1039/d3sc06357g (PMC10793598; doi:10.1039/d3sc06357g)
Supplement: SC-015-D3SC06357G-s003 [file SC-015-D3SC06357G-s003.pdf]

Electronic Supplementary Material (ESI) for Chemical Science.
This journal is © The Royal Society of Chemistry 2023

Electronic Supplementary Information

Redox-mediated electrochemiluminescence enhancement for bead-based immunoassay

Alessandro Fracassa,[a] Claudio Ignazio Santo,[a] Emily Kerr,[b] Sara Knezevic,[c] David J. Hayne,[b] Paul S. Francis,[d] Frederic Kanoufi,[e] Neso Sojic,[c] Francesco Paolucci[a] and Giovanni Valenti [a]

a. Department of Chemistry Giacomo Ciamician, University of Bologna, via Selmi 2, Bologna 40126, Italy. E-mail: g.valenti@unibo.it

b. Institute for Frontier Materials, Deakin University, Geelong, Victoria 3220, Australia.

c. Univ. Bordeaux, CNRS, Bordeaux INP, Institut des Sciences Moléculaires, UMR 5255, 33607 Pessac, France.

d. Deakin University, Centre for Sustainable Bioproducts, Faculty of Science, Engineering and Built Environment, Geelong, Victoria 3220, Australia.

e. Université Paris Cité, ITODYS, CNRS, F-75013 Paris, France.

Table of Contents

1 ECL microscopy 3

2 Photophysical and electrochemical characterisation of metal complexes 3

3 Reaction schemes 4

4 Photoelectrochemical characterisation of Ru@Beads and Ru@Beads/Ir 6

5 ECL images elaboration 9

6 Ru@Beads/$[Ir(sppy)_3]^{3-}$ ECL intensities at different concentrations of $[Ir(sppy)_3]^{3-}$ 14

7 Ru@Beads/$[Ir(sppy)_3]^{3-}$ current intensities at different concentrations of $[Ir(sppy)_3]^{3-}$ 15

8 Ru@Beads/$[Ir(sppy)_3]^{3-}$ ECL intensities at different concentrations of TPrA 17

9 Ru@Beads/$[Ir(dfppy)_2(pt\text{-}TEG)]^{+}$ ECL intensity at 1.5 V *vs* Ag/AgCl 24

10 Ru@Beads/$[Ir(bt)_2(pt\text{-}TEG)]^{+}$ ECL intensity at 1.3 V *vs* Ag/AgCl 25

11 Ru@Beads/$[Ir(bt)_2(pt\text{-}TEG)]^{+}$ ECL intensity at 1.2 V *vs* Ag/AgCl 28

12 Ru@Beads/$[Fe(bpy)_3]^{2+}$ ECL intensity at 1.2 V *vs* Ag/AgCl 30

13 Ir-mediated bead-based simulated ECL 32

13.1 General modelling strategy 32

13.2 Steady-state expression of the Ru ECL emission 32

13.2.1 Case of $[Ir(sppy)_3]^{3-}$ 33

13.2.2 Case of $[Ir(C^\wedge N)_2(pt\text{-}TEG)]^{+}$ 34

13.2.3 Case of $[Fe(bpy)_3]^{2+}$ 35

13.3 COMSOL simulation of the solution species concentration profiles 35

14 Collective beads experiment 39

15 References 41

1 ECL microscopy

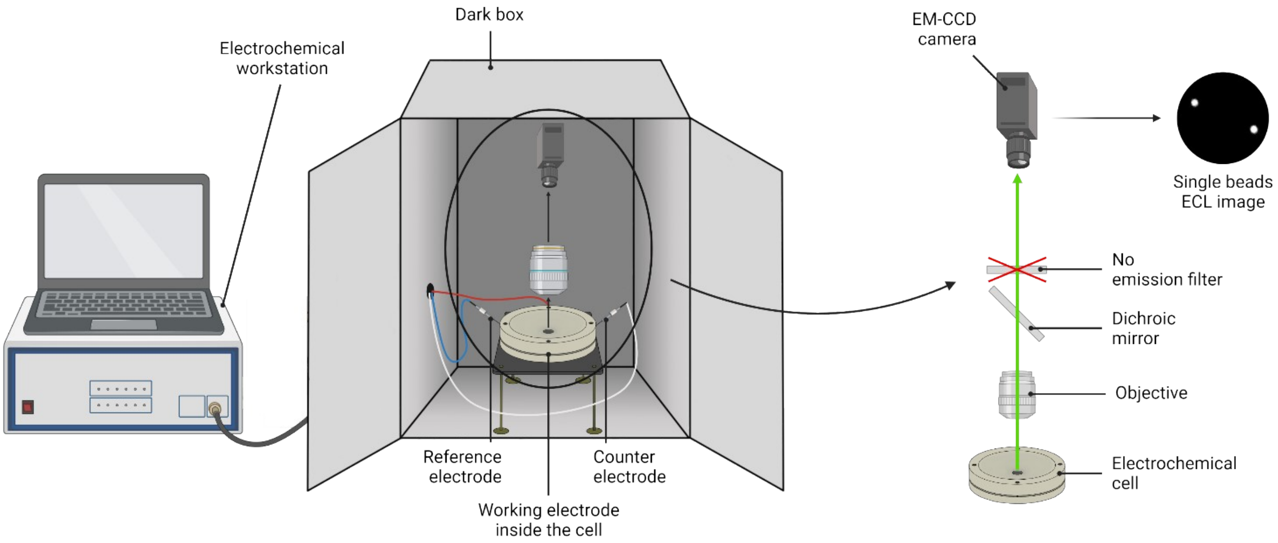

Figure S1. Experimental setup employed to record the ECL signal. The electrochemical workstation comprises a computer simultaneously connected to the digital camera and the BioLogic SP-300 potentiostat that controls the applied potential. The digital camera and the electrochemical cell were placed within a dark box that prevented the external light to be captured. The beads were injected in the electrochemical cell and attracted to the electrode surface by a magnet placed below the cell. Following the application of a suitable potential, ECL is produced, captured by the microscope objective and, eventually, it is transmitted directly to the EM-CCD camera without being filtered by any emission filter.

2 Photophysical and electrochemical characterisation of metal complexes

Metal complex	λ_{abs} (nm)	λ_{em} (nm λ_{exc} 340 nm)	E_{ox} (V *vs* Ag/AgCl)	E_{red} (V *vs* Ag/AgCl)
$[Ru(bpy)_3]^{2+}$	243, 285, 452	625	1.09	-1.31
$[Ir(sppy)_3]^{3-}$	245, 271, 360	515	0.83	-1.97
$[Ir(dfppy)_2(pt\text{-}TEG)]^{+}$	248, 312, 369	452, **481**	1.44	-1.71
$[Ir(bt)_2(pt\text{-}TEG)]^{+}$	323, 417	526, **562**	1.28	-1.66
$[Fe(bpy)_3]^{2+}$	-	-	0.85	-1.22

Table S1. Table summarising the photophysical and electrochemical properties of the investigated Ru(II), Ir(III) and Fe(II) complexes. All the listed data are retrieved from the literature.[1–5]

3 Reaction schemes

$$TPrA \rightarrow TPrA^{\bullet +} + e^{-} \quad (1.1)$$

$$TPrA^{\bullet +} \rightarrow TPrA^{\bullet} + H^{+} \quad (1.2)$$

$$[Ru(bpy)_3]^{2+} + TPrA^{\bullet} \rightarrow [Ru(bpy)_3]^{+} + Im^{+} \quad (1.3)$$

$$[Ru(bpy)_3]^{+} + TPrA^{\bullet +} \rightarrow [Ru(bpy)_3]^{2+*} + TPrA \quad (1.4)$$

$$[Ru(bpy)_3]^{2+*} \rightarrow [Ru(bpy)_3]^{2+} + h\nu \; (620\; nm) \quad (1.5)$$

Scheme S1. Heterogeneous ECL reaction mechanism between $[Ru(bpy)_3]^{2+}$ and TPrA that occurs, for example, when the luminophore is constrained to the surface of magnetic beads in commercial immunoassay and cannot be directly oxidised at the electrode surface.

$$TPrA \rightarrow TPrA^{\bullet +} + e^{-} \quad (2.1)$$

$$[Ir(sppy)_3]^{3-} \rightarrow [Ir(sppy)_3]^{2-} + e^{-} \quad (2.2)$$

$$[Ir(sppy)_3]^{3-} + TPrA^{\bullet +} \rightleftharpoons [Ir(sppy)_3]^{2-} + TPrA \quad (2.3)$$

$$TPrA^{\bullet +} \rightarrow TPrA^{\bullet} + H^{+} \quad (2.4)$$

$$[Ir(sppy)_3]^{2-} + TPrA^{\bullet} \rightarrow [Ir(sppy)_3]^{3-*} + Im^{+} \quad (2.5)$$

$$[Ir(sppy)_3]^{3-*} \rightarrow [Ir(sppy)_3]^{3-} + h\nu \; (515\; nm) \quad (2.6)$$

$$[Ru(bpy)_3]^{2+} + TPrA^{\bullet} \rightarrow [Ru(bpy)_3]^{+} + Im^{+} \quad (2.7)$$

$$[Ru(bpy)_3]^{+} + [Ir(sppy)_3]^{2-} \rightarrow [Ru(bpy)_3]^{2+*} + [Ir(sppy)_3]^{3-} \quad (2.8)$$

$$[Ru(bpy)_3]^{+} + TPrA^{\bullet +} \rightarrow [Ru(bpy)_3]^{2+*} + TPrA \quad (2.9)$$

$$[Ru(bpy)_3]^{2+*} \rightarrow [Ru(bpy)_3]^{2+} + h\nu \; (620\; nm) \quad (2.10)$$

Scheme S2. Photophysically and energetically feasible reactions between $[Ru(bpy)_3]^{2+}$, $[Ir(sppy)_3]^{3-}$ and TPrA reaction partners.

$$TPrA \rightarrow TPrA^{\bullet +} + e^{-} \quad (3.1)$$

$$[Ir(dfppy)_2(pt\text{-}TEG)]^{+} \rightarrow [Ir(dfppy)_2(pt\text{-}TEG)]^{2+} + e^{-} \quad (3.2)$$

$$[Ir(dfppy)_2(pt\text{-}TEG)]^{2+} + TPrA \rightarrow [Ir(dfppy)_2(pt\text{-}TEG)]^{+} + TPrA^{\bullet +} \quad (3.3)$$

$$TPrA^{\bullet +} \rightarrow TPrA^{\bullet} + H^{+} \quad (3.4)$$

$$[Ir(dfppy)_2(pt\text{-}TEG)]^{+} + TPrA^{\bullet} \rightarrow [Ir(dfppy)_2(pt\text{-}TEG)] + Im^{+} \quad (3.5)$$

$$[Ir(dfppy)_2(pt\text{-}TEG)] + TPrA^{\bullet +} \rightarrow [Ir(dfppy)_2(pt\text{-}TEG)]^{+} + TPrA \quad (3.6)$$

$$[Ir(dfppy)_2(pt\text{-}TEG)]^{2+} + TPrA^{\bullet} \rightarrow [Ir(dfppy)_2(pt\text{-}TEG)]^{+*} + Im^{+} \quad (3.7)$$

$$[Ir(dfppy)_2(pt\text{-}TEG)]^{+*} \rightarrow [Ir(dfppy)_2(pt\text{-}TEG)]^{+} + h\nu \ (480\ nm) \quad (3.8)$$

$$[Ru(bpy)_3]^{2+} + TPrA^{\bullet} \rightarrow [Ru(bpy)_3]^{+} + Im^{+} \quad (3.9)$$

$$[Ru(bpy)_3]^{+} + TPrA^{\bullet +} \rightarrow [Ru(bpy)_3]^{2+*} + TPrA \quad (3.10)$$

$$[Ru(bpy)_3]^{2+} + [Ir(dfppy)_2(pt\text{-}TEG)] \rightarrow [Ru(bpy)_3]^{+} + [Ir(dfppy)_2(pt\text{-}TEG)]^{+} \quad (3.11)$$

$$[Ru(bpy)_3]^{2+*} \rightarrow [Ru(bpy)_3]^{2+} + h\nu \ (620\ nm) \quad (3.12)$$

Scheme S3. Photophysically and energetically feasible reactions between $[Ru(bpy)_3]^{2+}$, $[Ir(dfppy)_2(pt\text{-}TEG)]^{+}$ and TPrA reaction partners.

$$TPrA \rightarrow TPrA^{\bullet +} + e^{-} \quad (4.1)$$

$$[Ir(bt)_2(pt\text{-}TEG)]^{+} \rightarrow [Ir(bt)_2(pt\text{-}TEG)]^{2+} + e^{-} \quad (4.2)$$

$$[Ir(bt)_2(pt\text{-}TEG)]^{2+} + TPrA \rightarrow [Ir(bt)_2(pt\text{-}TEG)]^{+} + TPrA^{\bullet +} \quad (4.3)$$

$$TPrA^{\bullet +} \rightarrow TPrA^{\bullet} + H^{+} \quad (4.4)$$

$$[Ir(bt)_2(pt\text{-}TEG)]^{+} + TPrA^{\bullet} \rightarrow [Ir(bt)_2(pt\text{-}TEG)] + Im^{+} \quad (4.5)$$

$$[Ir(bt)_2(pt\text{-}TEG)] + TPrA^{\bullet +} \rightarrow [Ir(bt)_2(pt\text{-}TEG)]^{+*} + TPrA \quad (4.6)$$

$$[Ir(bt)_2(pt\text{-}TEG)]^{2+} + TPrA^{\bullet} \rightarrow [Ir(bt)_2(pt\text{-}TEG)]^{+*} + Im^{+} \quad (4.7)$$

$$[Ir(bt)_2(pt\text{-}TEG)]^{+*} \rightarrow [Ir(bt)_2(pt\text{-}TEG)]^{+} + h\nu \ (560\ nm) \quad (4.8)$$

$$[Ru(bpy)_3]^{2+} + TPrA^{\bullet} \rightarrow [Ru(bpy)_3]^{+} + Im^{+} \quad (4.9)$$

$$[Ru(bpy)_3]^{+} + TPrA^{\bullet +} \rightarrow [Ru(bpy)_3]^{2+*} + TPrA \quad (4.10)$$

$$[Ru(bpy)_3]^{2+} + [Ir(bt)_2(pt\text{-}TEG)] \rightarrow [Ru(bpy)_3]^{+} + [Ir(bt)_2(pt\text{-}TEG)]^{+} \quad (4.11)$$

$$[Ru(bpy)_3]^{2+*} \rightarrow [Ru(bpy)_3]^{2+} + h\nu \ (620\ nm) \quad (4.12)$$

Scheme S4. Photophysically and energetically feasible reactions between $[Ru(bpy)_3]^{2+}$, $[Ir(bt)_2(pt\text{-}TEG)]^{+}$ and TPrA reaction partners.

4 Photoelectrochemical characterisation of Ru@Beads and Ru@Beads/Ir

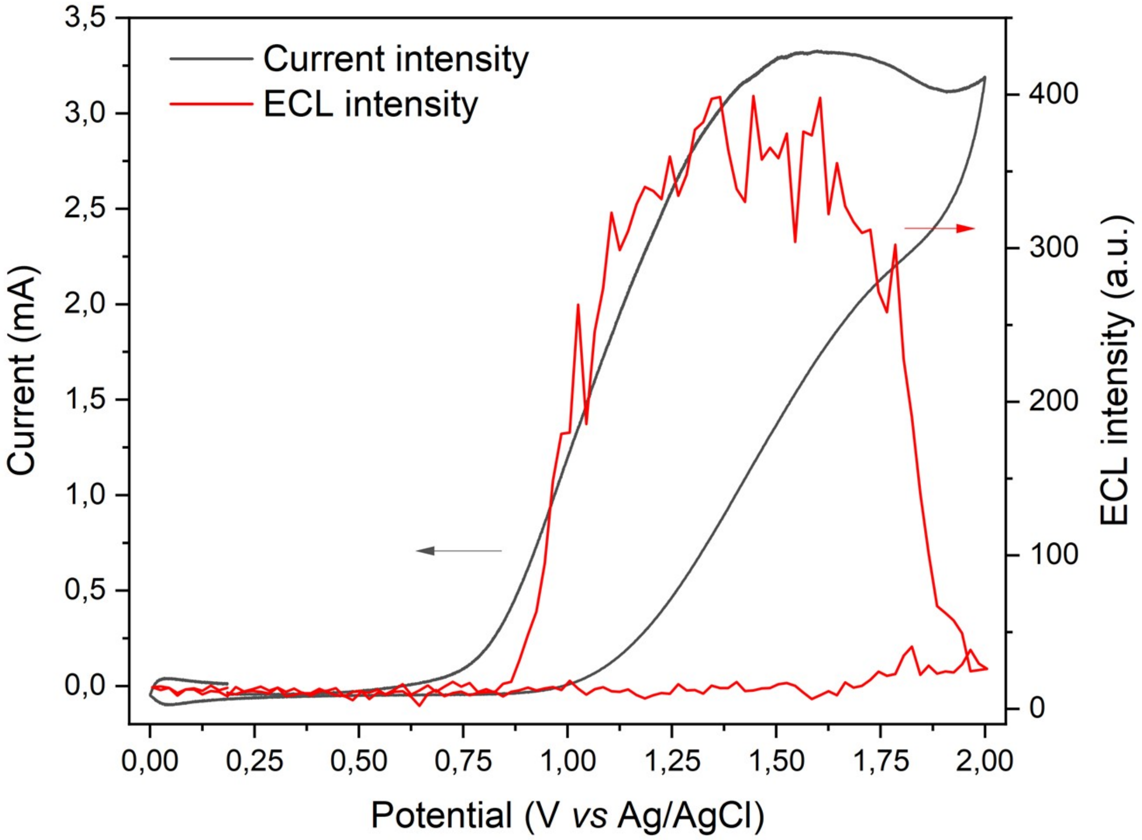

Figure S2. CV-ECL measurement performed on Ru@Beads in a 0.3 M PB solution at pH 6.8 with 180 mM TPrA. The working electrode potential was scanned at 100 mV/s from OCP up to 2 V (*vs* Ag/AgCl), back to 0 V (*vs* Ag/AgCl) and, eventually, terminating the cycle at OCP. The beads ECL emission (red line) was acquired each 200 ms and, for each frame, the maximum value of the beads ECL profile is plotted versus the applied potential. The background signal is eliminated by subtracting, for each frame, the average ECL intensity value of the background retrieved over a 50x50 pixel square centred in a region of the image where no beads are present.

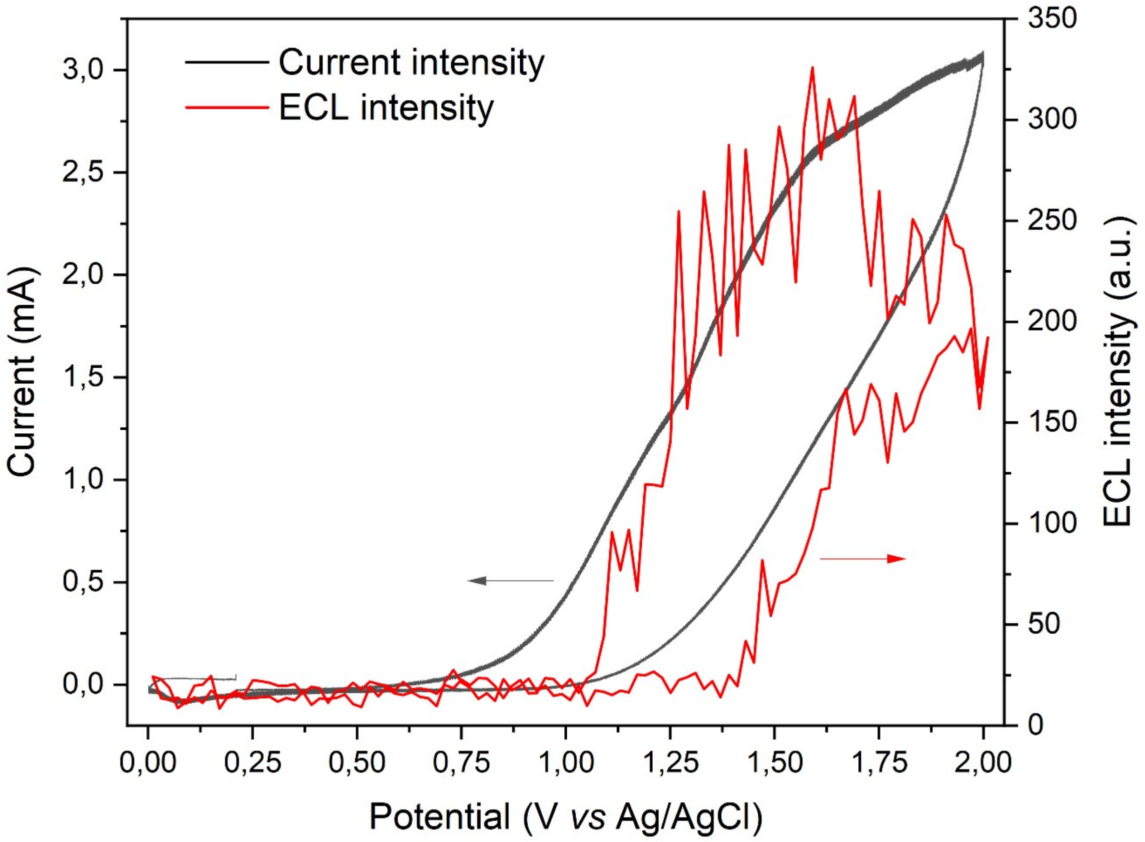

Figure S3. CV-ECL measurement performed on Ru@Beads in a 0.3 M PB solution at pH 6.8 with 180 mM TPrA and 100 µM $[Ir(dfppy)_2(pt\text{-}TEG)]^+$. The working electrode potential was scanned at 100 mV/s from OCP up to 2 V (*vs* Ag/AgCl), back to 0 V (*vs* Ag/AgCl) and, eventually, terminating the cycle at OCP. The beads ECL emission (red line) was acquired each 200 ms and, for each frame, the maximum value of the beads ECL profile is plotted versus the applied potential. The background signal is eliminated by subtracting, for each frame, the average ECL intensity value of the background retrieved over a 50x50 pixel square centred in a region of the image where no beads are present.

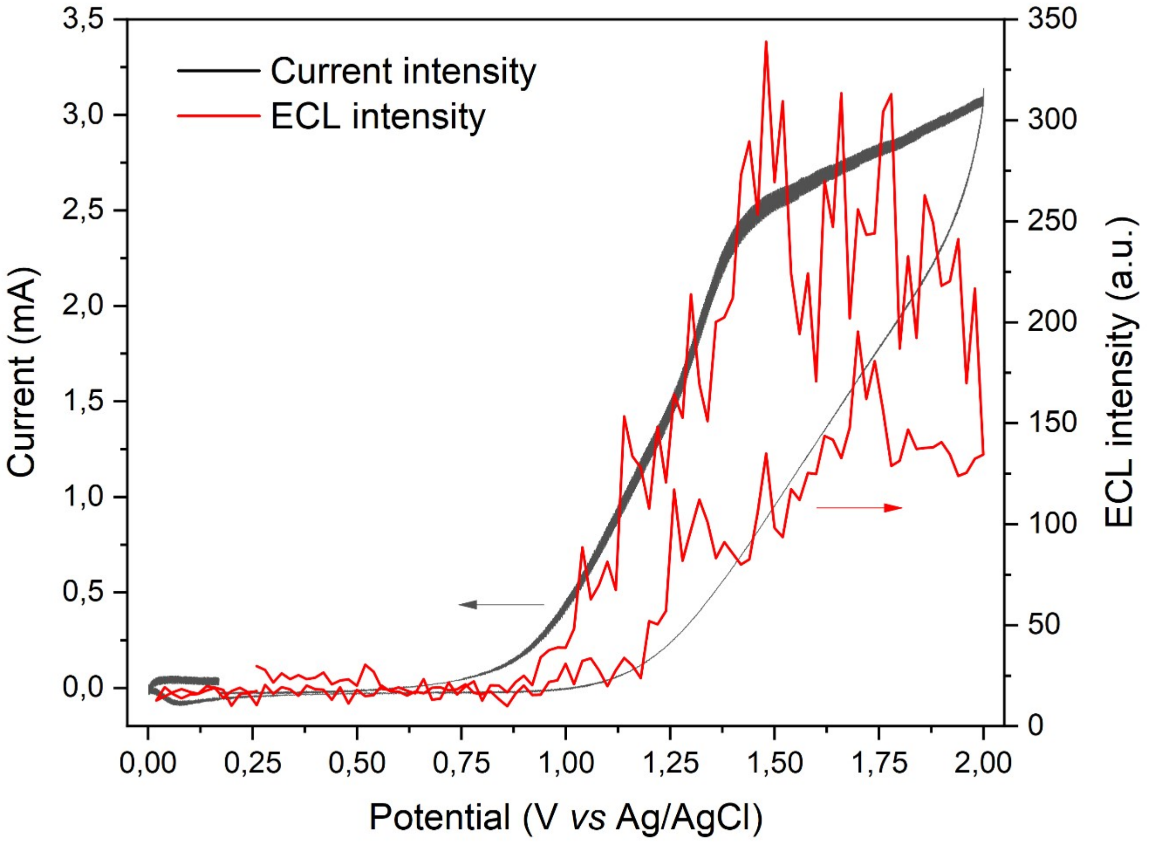

Figure S4. CV-ECL measurement performed on Ru@Beads in a 0.3 M PB solution at pH 6.8 with 180 mM TPrA and 100 µM $[Ir(bt)_2(pt\text{-}TEG)]^+$. The working electrode potential was scanned at 100 mV/s from OCP up to 2 V (*vs* Ag/AgCl), back to 0 V (*vs* Ag/AgCl) and, eventually, terminating the cycle at OCP. The beads ECL emission (red line) was acquired each 200 ms and, for each frame, the maximum value of the beads ECL profile is plotted versus the applied potential. The background signal is eliminated by subtracting, for each frame, the average ECL intensity value of the background retrieved over a 50x50 pixel square centred in a region of the image where no beads are present.

5 ECL images elaboration

The ECL intensity profiles were extracted using the default profile settings of the ImageJ software from a 90x4 pixel rectangle (14.28x0.63 µm) centred on the bead. Thus, the software generates a profile where the signal intensity for each pixel along the abscissae (i.e., distance) is averaged over the signal intensity of 4 adjacent pixels that lie at the same distance. To consider only the beads emission (i.e., the emission from $[Ru(bpy)_3]^{2+}$ labels), the background signal was subtracted to the respective ECL profile. The background emission intensity could, in principle, be determined by averaging the ECL intensity of the first 10 pixels (1.59 µm) where no bead emission is detected (Fig. S5).

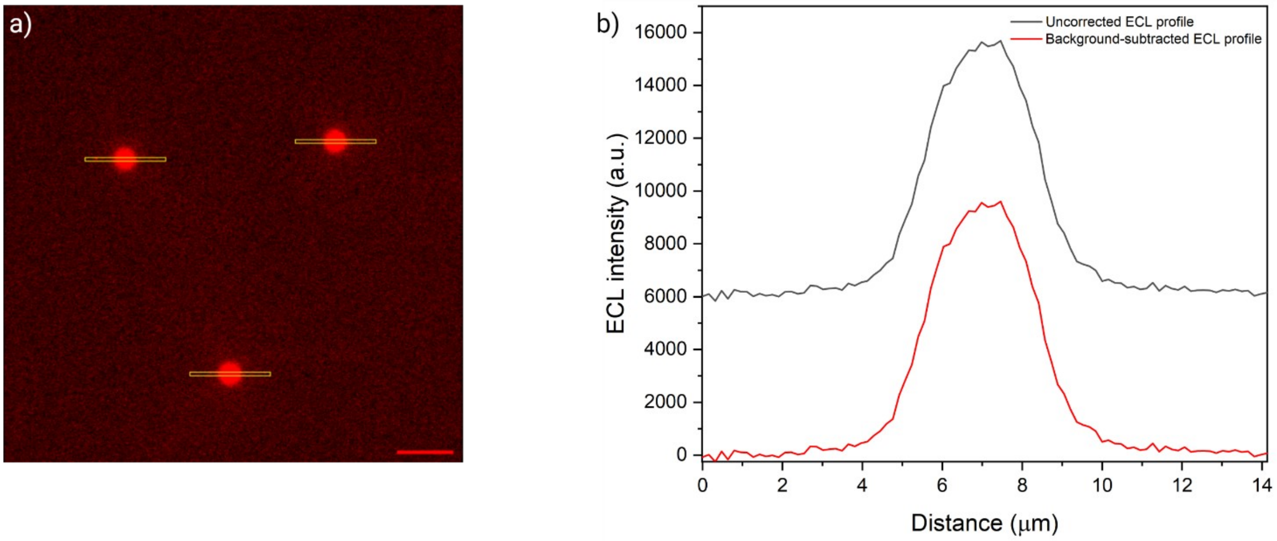

Figure S5. (a) ECL image of 2.8 µm single-beads covalently labelled with $[Ru(bpy)_3]^{2+}$ in a 0.3 M PB solution at pH 6.8 with 180 mM TPrA and 100 µM of $[Ir(dfppy)_2(pt\text{-}TEG)]^{+}$. The image was captured with an EM-CCD camera by recording the ECL signal for 10 s during a two-step chronoamperometry measurement: 2 s at OCP and 8 s at 1.5 V vs Ag/AgCl. Magnification, X100; objective numerical aperture, 0.8; gain, 1; sensitivity, 255; contrast scale, 5000 to 11000; scale bar, 10 µm. The image includes 90x4 pixel rectangles (highlighted in yellow) employed for plotting the uncorrected ECL profile (b, grey line). The background subtracted ECL profile (b, red line) was obtained by subtracting the average value of the first 10 pixels, where no bead emission is detected.

To verify the validity of the aforementioned approach for subtracting the background signal, ECL images of streptavidin-coated beads (i.e., not labelled with $[Ru(bpy)_3]^{2+}$) were captured in analogous conditions to the respective Ru@Beads/Ir system (Beads/Ir, Fig. S6b). The ECL profile obtained from a given Beads/Ir system was exploited as the baseline that needed to be subtracted from the uncorrected profile of the respective Ru@Beads/Ir system (i.e., the raw ECL profile including the background signal, Fig. S6c). Eventually, to ascertain any differences, we compared the elaborated ECL profiles processed in both manners, namely by subtracting the average ECL signal of the first 10 pixel and by subtracting the ECL profile of the respective Beads/Ir system (Fig. S6d). For Beads/$[Ir(sppy)_3]^{3-}$ at 100 µM of $[Ir(sppy)_3]^{3-}$ and at any concentration of TPrA, the presence of 2.8 µm beads does not affect the ECL signal recorded by the EM-CCD camera which is constant for the whole image. Hence, we postulated that subtracting the average ECL intensity of the initial 10 pixel is a valid processing strategy for the experiments conducted at any other concentration of $[Ir(sppy)_3]^{3-}$.

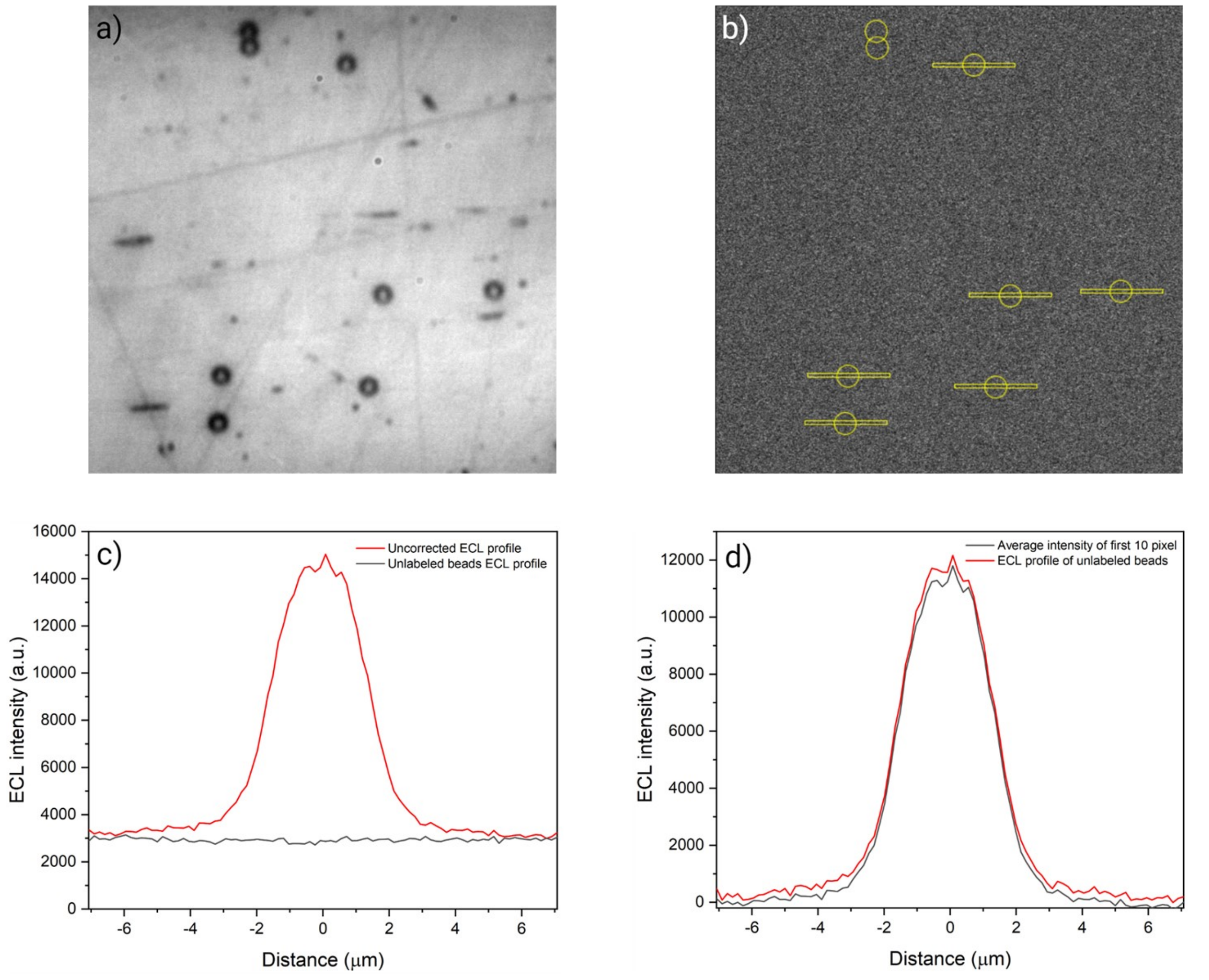

Figure S6. (a) Optical image of 2.8 µm single-beads coated with streptavidin in 0.3 M PB at pH 6.8 with 180 mM TPrA and 100 µM of $[Ir(sppy)_3]^{3-}$. The image was captured with a CCD camera for a total integration time of 200 ms. Magnification, X100; objective numerical aperture, 0.8; contrast scale, automatically adjusted by ImageJ at 5846 to 8767. (b) ECL image of the same system, captured with an EM-CCD camera by recording the ECL signal for 10 s during a two-step chronoamperometry measurement: 2 s at OCP and 8 s at 1.2 V *vs* Ag/AgCl. Magnification, X100; objective numerical aperture, 0.8; gain, 1; sensitivity, 255; contrast scale, automatically adjusted by ImageJ at 1839 to 5100. The image includes round shapes that indicate the beads position and 90x4 pixel rectangles employed for plotting the ECL profile (c, grey line) which is meant to be the baseline of Ru@Beads/$[Ir(sppy)_3]^{3-}$ uncorrected ECL profile (c, red line). (d) A comparison between the ECL profiles, both belonging to the Ru@Beads/$[Ir(sppy)_3]^{3-}$system, corrected by applying two different approaches: by subtracting to each point the average value of the first 10 pixels (grey line) and by subtracting the ECL profile of Beads/$[Ir(sppy)_3]^{3-}$ (red line).

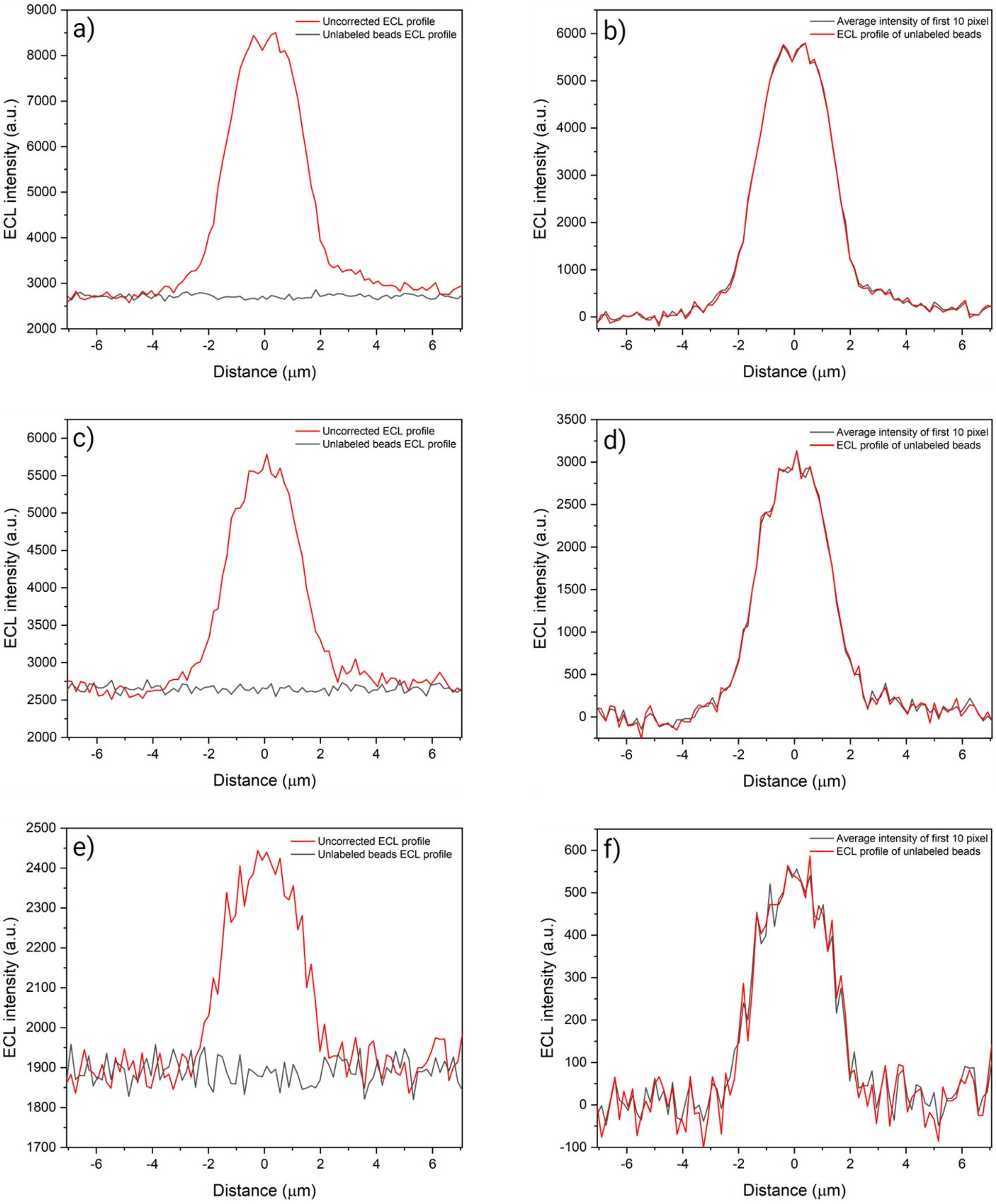

Figure S7. Comparative approach to validate the background subtraction processes for the following systems: (a,b) 2.8 µm single-beads in 0.3 M PB at pH 6.8 with 100 mM TPrA and 100 µM of $[Ir(sppy)_3]^{3-}$, (c,d) 2.8 µm single-beads in 0.3 M PB at pH 6.8 with 50 mM TPrA and 100 µM of $[Ir(sppy)_3]^{3-}$, (e,f) 2.8 µm single-beads in 0.3 M PB at pH 6.8 with 25 mM TPrA and 100 µM of $[Ir(sppy)_3]^{3-}$. The ECL profiles were obtained from ECL images captured using an EM-CCD camera during a two-step chronoamperometry measurement: 2 s at OCP and 8 s at 1.2 V *vs* Ag/AgCl. Magnification, X100; objective numerical aperture, 0.8; gain, 1; sensitivity, 350. The ECL profiles obtained by subtracting the average ECL intensity of the initial 10 pixel (b, d, f, grey line) are compared to the ECL profiles determined with the alternative method (b, d, f, red line), namely by subtracting the ECL profile of a given Beads/$[Ir(sppy)_3]^{3-}$ system (a, c, e, grey line) to the non-processed ECL profile collected from the respective Ru@Beads/$[Ir(sppy)_3]^{3-}$ system (a, c, e, red line).

The same approach was then employed for every other system where the mediated species emits in the background. For Beads/[Ir(dfppy)$_2$(pt-TEG)]$^+$ at 1.5 V *vs* Ag/AgCl, the presence of 2.8 µm beads does not affect the ECL signal recorded by the EM-CCD camera which is constant for the whole image (Fig. S8). While, for Ru@Beads/[Ir(dfppy)$_2$(pt-TEG)]$^+$ at 1.2 V vs Ag/AgCl, the Ir(III) species is not directly oxidised and the energetics of the system prevents its excitation.

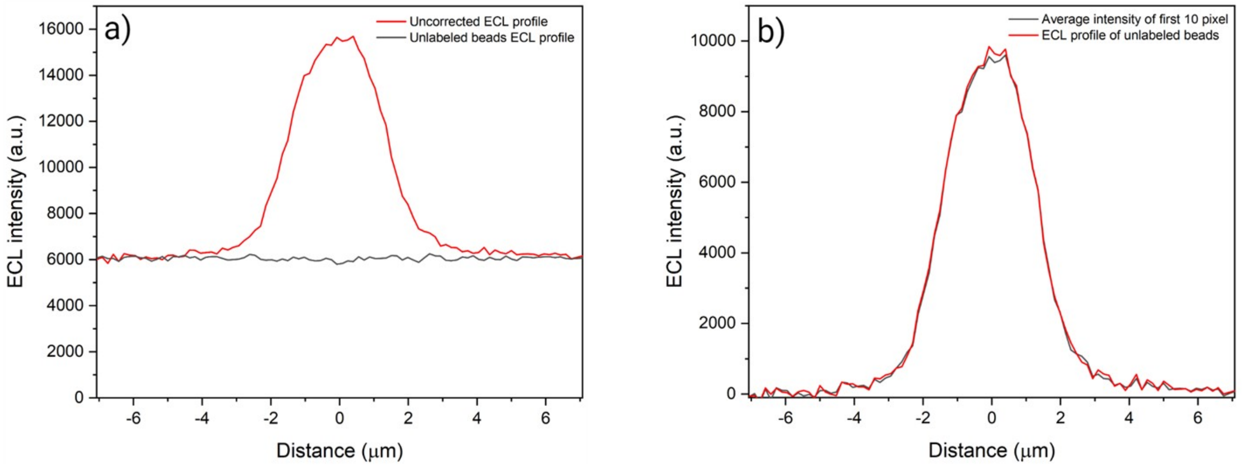

Figure S8. Comparative approach to validate the background subtraction processes for 2.8 µm single-beads in 0.3 M PB at pH 6.8 with 180 mM TPrA and 100 µM of [Ir(dfppy)$_2$(pt-TEG)]$^+$. The ECL profiles were obtained from ECL images captured using an EM-CCD camera during a two-step chronoamperometry measurement: 2 s at OCP and 8 s at 1.5 V *vs* Ag/AgCl. Magnification, X100; objective numerical aperture, 0.8; gain, 1; sensitivity, 255. The ECL profile obtained by subtracting the average ECL intensity of the initial 10 pixel (b, grey line) is compared to the ECL profile determined with the alternative method (b, red line), namely by subtracting the ECL profile of Beads/[Ir(dfppy)$_2$(pt-TEG)]$^+$ (a, grey line) to the non-processed ECL profile collected from Ru@Beads/[Ir(dfppy)$_2$(pt-TEG)]$^+$ (a, red line).

The outcome is different with Beads/[Ir(bt)$_2$(pt-TEG)]$^+$ where the microbeads prevent the emitting species from diffusing to the top of the microspheres (Fig. S9). Thus, the ECL profiles of Beads/[Ir(bt)$_2$(pt-TEG)]$^+$ recorded at 1.2 V and 1.3 V *vs* Ag/AgCl are subtracted to the Ru@Beads/[Ir(bt)$_2$(pt-TEG)]$^+$ ECL profiles at the respective potentials in order to allow an accurate interpretation of the reaction mechanism. This elaboration strategy was not adopted for CV-ECL measurement on Ru@Beads/[Ir(bt)$_2$(pt-TEG)]$^+$ and for integrated measurements on the same system at 10 and 50 µM concentration of [Ir(bt)$_2$(pt-TEG)]$^+$. We decided to apply this approach since, in this research, the CV-ECL measurements are employed only qualitatively, while the trend established within integrated measurements at different concentration of [Ir(bt)$_2$(pt-TEG)]$^+$ (Fig. S22-S23) is still preserved since the ECL signal is just slightly underestimated.

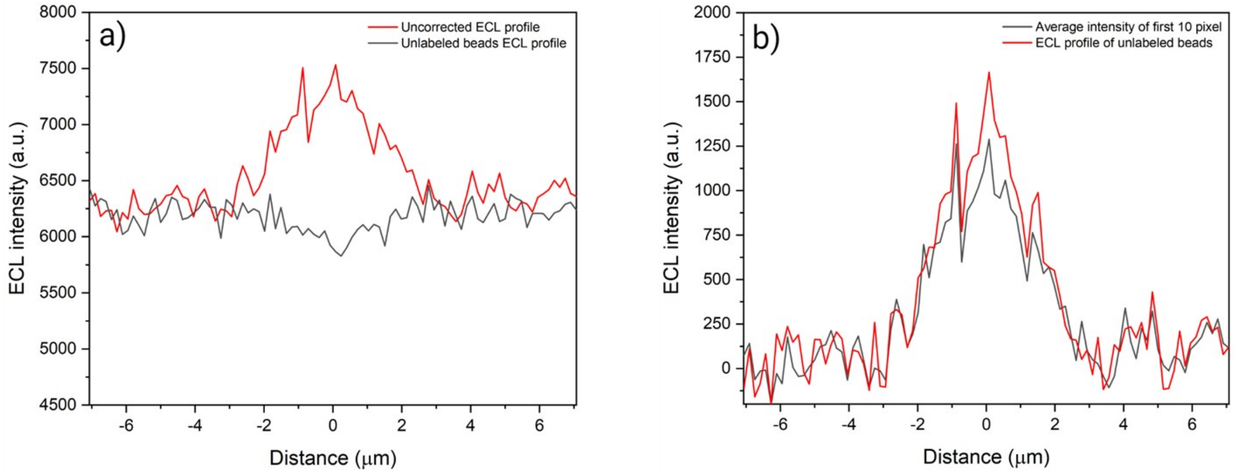

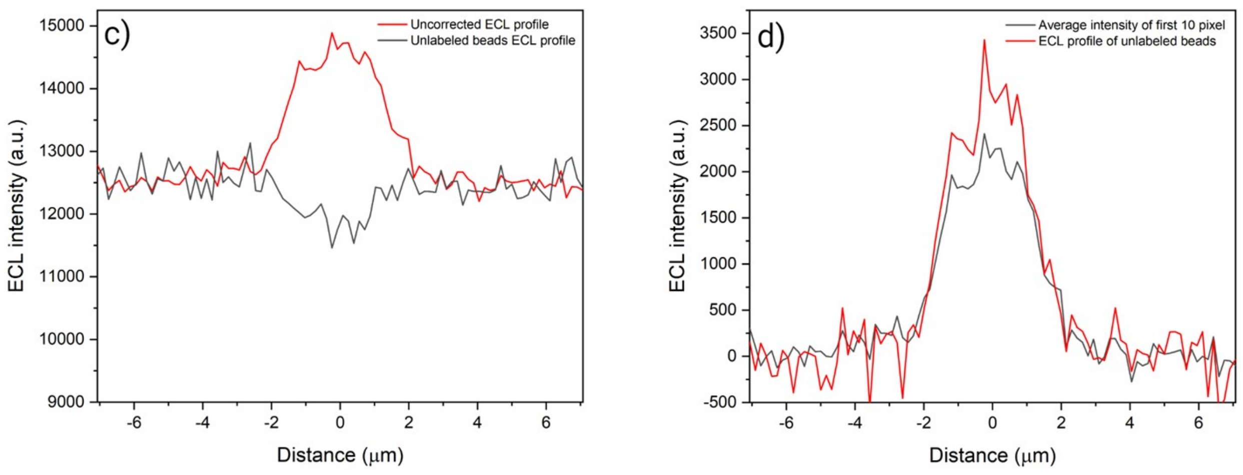

Figure S9. Comparative approach to validate the background subtraction processes for 2.8 μm single-beads in 0.3 M PB at pH 6.8 with 180 mM TPrA and 100 μM of [Ir(bt)$_2$(pt-TEG)]$^+$. The ECL profiles were obtained from ECL images captured using an EM-CCD camera during a two-step chronoamperometry measurement: 2 s at OCP and 8 s at 1.2 V *vs* Ag/AgCl (Fig. a, b) and 2 s at OCP and 8 s at 1.3 V *vs* Ag/AgCl (Fig. c, d). Magnification, X100; objective numerical aperture, 0.8; gain, 1; sensitivity, 255. The ECL profiles obtained by subtracting the average ECL intensity of the initial 10 pixel (b, d, grey line) are compared to the ECL profiles determined with the alternative method (b, d, red line), namely by subtracting the ECL profile of a given Beads/[Ir(bt)$_2$(pt-TEG)]$^+$ system (a, c, grey line) to the non-processed ECL profile collected from the respective Ru@Beads/[Ir(bt)$_2$(pt-TEG)]$^+$ system (a, c, red line).

Eventually, for Ru@Beads/[Fe(bpy)$_3$]$^{2+}$, the background signal is determined as the average of the ECL intensity of the first 10 pixels since [Fe(bpy)$_3$]$^{2+}$ is a non-emitting complex.
False colour was applied to all the integrated ECL images using Red Lookup Table in ImageJ.
The ECL intensity within CV-ECL images is obtained by plotting, for each frame, the maximum value of the ECL profile that is extracted from a 90x4 pixel rectangle centred on the bead. The ECL signal is averaged over all the beads present in the respective frame. The background signal is eliminated by subtracting to the maximum ECL intensity value of the emission profile, for each frame, the average ECL intensity value of the background retrieved over a 50x50 pixel square.

6 Ru@Beads/$[Ir(sppy)_3]^{3-}$ ECL intensities at different concentrations of $[Ir(sppy)_3]^{3-}$

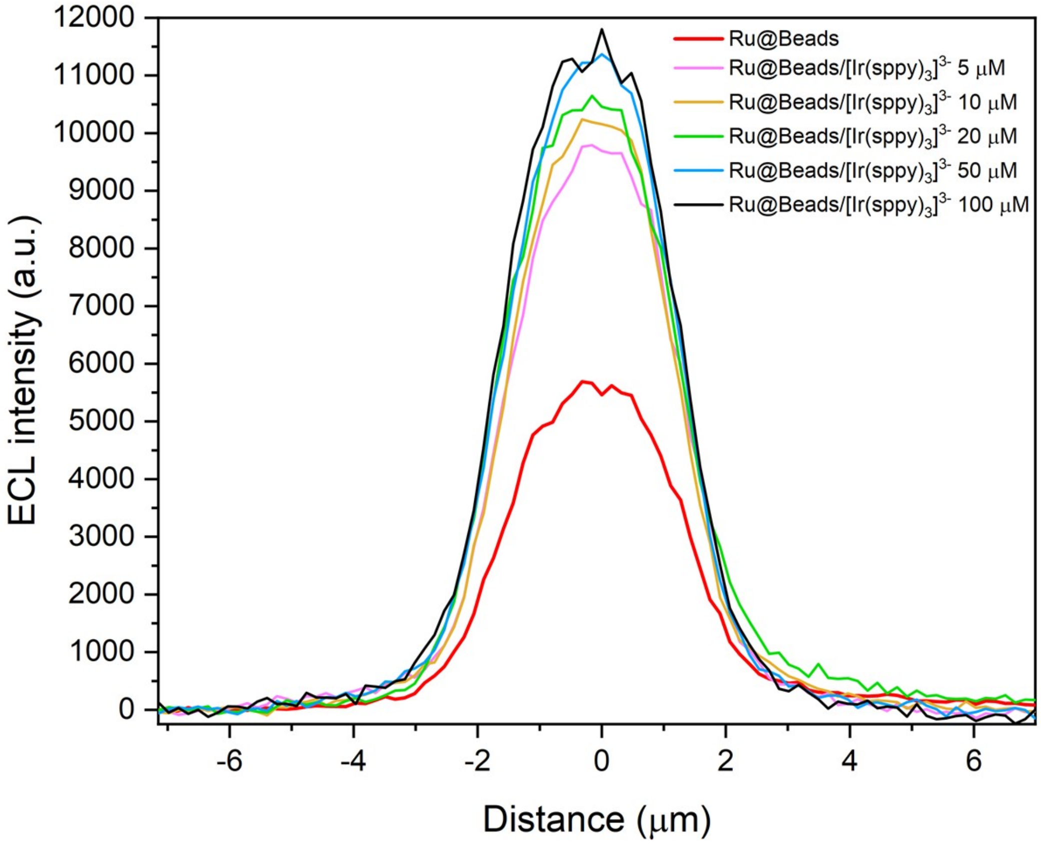

Figure S10. Comparison between ECL profiles of Ru@Beads (red line) and of Ru@Beads/$[Ir(sppy)_3]^{3-}$ in a 0.3 M PB solution at pH 6.8 with 180 mM TPrA and different $[Ir(sppy)_3]^{3-}$ concentrations: 5 μM (pink line), 10 μM (yellow line), 20 μM (green line), 50 μM (blue line) and 100 μM (black line). The ECL profiles were obtained from ECL images captured using an EM-CCD camera during a two-step chronoamperometry measurement: 2 s at OCP and 8 s at 1.2 V *vs* Ag/AgCl. Magnification, X100; objective numerical aperture, 0.8; gain, 1; sensitivity, 255. The profiles were extracted from 90x4 pixel rectangles centred on the beads and, eventually, processed by subtracting the background signal as the average emission intensity of the initial 10 pixel. The background emission arises from homogeneous ECL. Data are averaged over a minimum of six beads ($n \geq 6$).

7 Ru@Beads/$[Ir(sppy)_3]^{3-}$ current intensities at different concentrations of $[Ir(sppy)_3]^{3-}$

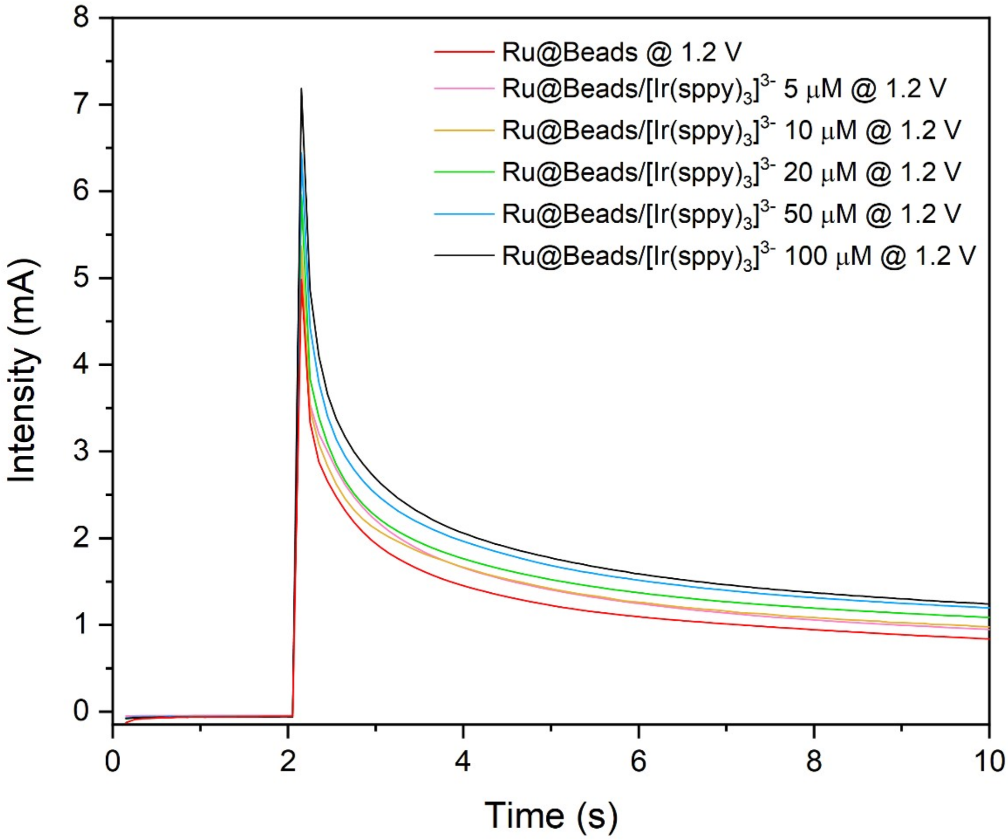

Figure S11. Comparison between current intensities of Ru@Beads (red line) and of Ru@Beads/$[Ir(sppy)_3]^{3-}$ in a 0.3 M PB solution at pH 6.8 with 180 mM TPrA and different $[Ir(sppy)_3]^{3-}$ concentrations: 5 µM (pink line), 10 µM (yellow line), 20 µM (green line), 50 µM (blue line) and 100 µM (black line). The current signals of each system were collected during a two-step chronoamperometry measurement: 2 s at open circuit potential (OCP) and 8 s at 1.2 V *vs* Ag/AgCl.

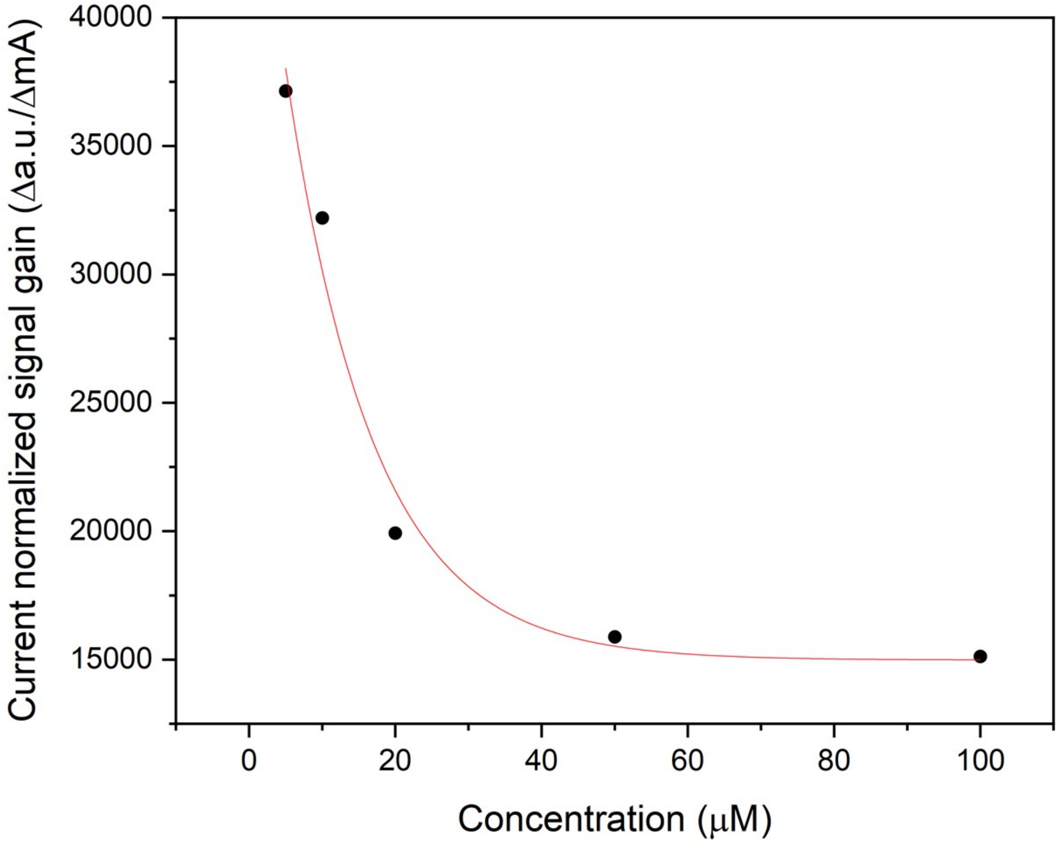

Figure S12. Comparison between current-normalised ECL signal gain of Ru@Beads/$[Ir(sppy)_3]^{3-}$ obtained at different $[Ir(sppy)_3]^{3-}$ concentrations: 5 µM, 10 µM, 20 µM, 50 µM and 100 µM. Each point is computed by dividing the ECL signal gain (i.e., the difference determined by subtracting the maximum ECL intensity of the Ru@Beads profile to that of the Ru@Beads/$[Ir(sppy)_3]^{3-}$ profile) by the current signal gain (i.e., the difference determined by subtracting the current intensity of Ru@Beads recorded at the 10th second to that of Ru@Beads/$[Ir(sppy)_3]^{3-}$ recorded at the 10th second) for the respective system. The red line represents the fitting function.

8 Ru@Beads/$[Ir(sppy)_3]^{3-}$ ECL intensities at different concentrations of TPrA

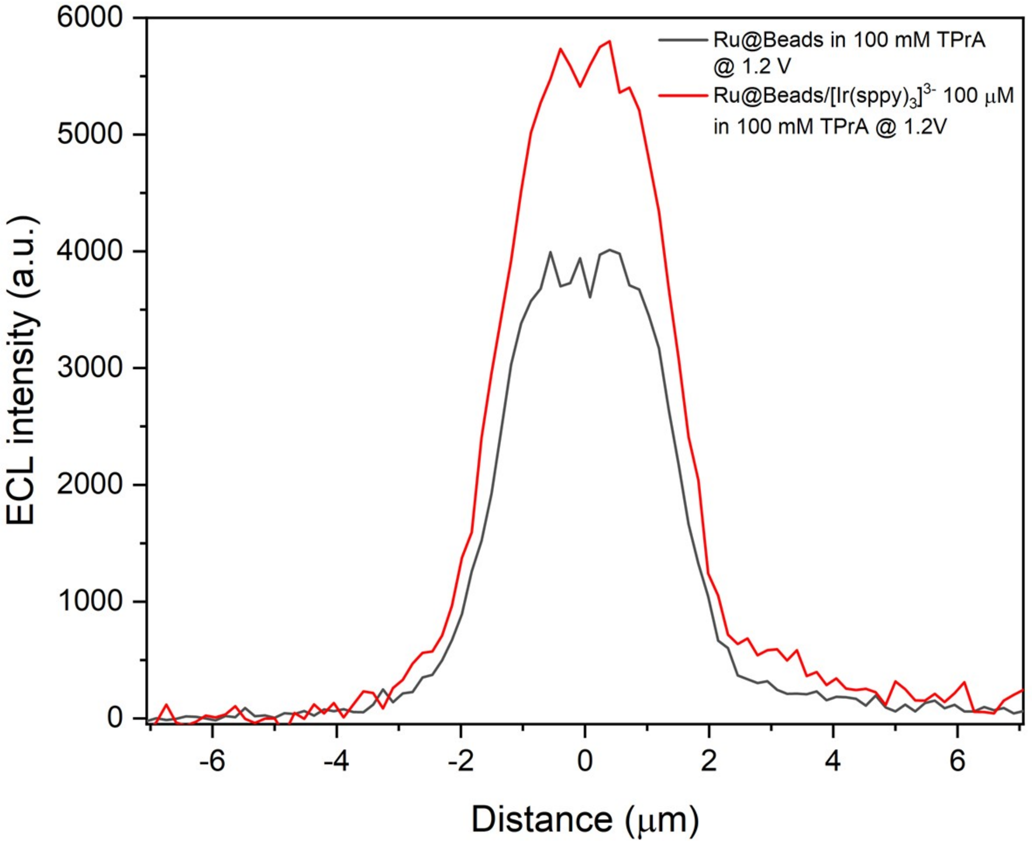

Figure S13. Comparison between ECL profiles of Ru@Beads (grey line) and of Ru@Beads/$[Ir(sppy)_3]^{3-}$ at 100 μM concentration of $[Ir(sppy)_3]^{3-}$ (red line) in a 0.3 M PB solution at pH 6.8 with 100 mM TPrA. The ECL profiles were obtained from ECL images captured using an EM-CCD camera during a two-step chronoamperometry measurement: 2 s at OCP and 8 s at 1.2 V *vs* Ag/AgCl. Magnification, X100; objective numerical aperture, 0.8; gain, 1; sensitivity, 350. The profiles were extracted from 90x4 pixel rectangles centred on the beads and, eventually, processed by subtracting the background signal as the average emission intensity of the initial 10 pixel. The background emission arises from homogeneous ECL. Data are averaged over a minimum of six beads (n ≥ 6).

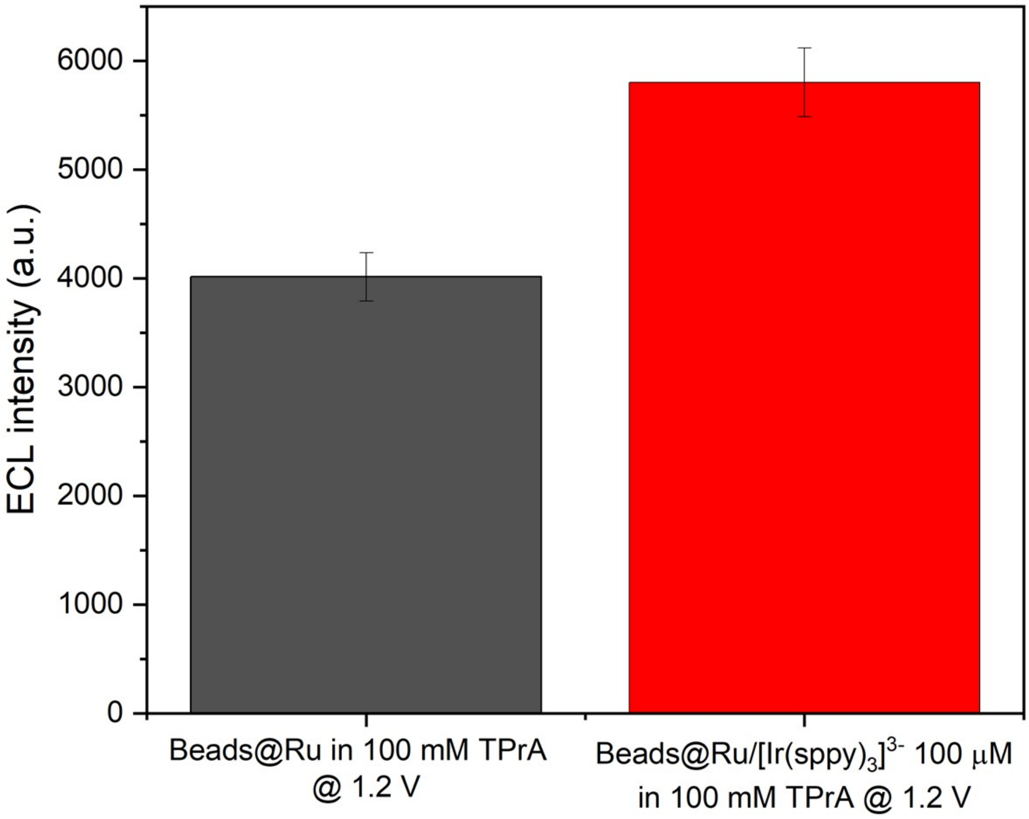

Figure S14. Comparison between ECL intensities of Ru@Beads (grey bar) and of Ru@Beads/$[Ir(sppy)_3]^{3-}$ at 100 µM concentration of $[Ir(sppy)_3]^{3-}$ (red bar) in a 0.3 M PB solution at pH 6.8 with 100 mM TPrA. The ECL intensities were obtained from ECL images captured using an EM-CCD camera during a two-step chronoamperometry measurement: 2 s at OCP and 8 s at 1.2 V *vs* Ag/AgCl. Magnification, X100; objective numerical aperture, 0.8; gain, 1; sensitivity, 350. Data are averaged over a minimum of six beads ($n \geq 6$). Each bar represents the maximum value of the respective ECL profile and the error bars show the standard errors.

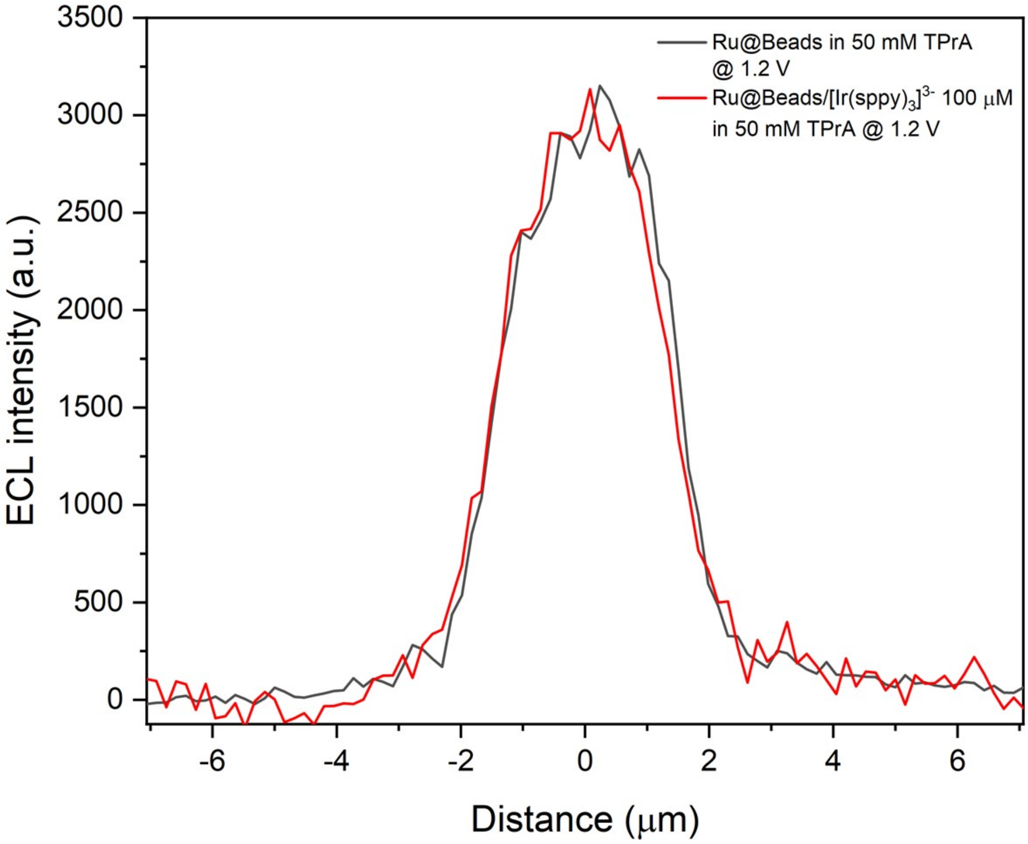

Figure S15. Comparison between ECL profiles of Ru@Beads (grey line) and of Ru@Beads/$[Ir(sppy)_3]^{3-}$ at 100 µM concentration of $[Ir(sppy)_3]^{3-}$ (red line) in a 0.3 M PB solution at pH 6.8 with 50 mM TPrA. The ECL profiles were obtained from ECL images captured using an EM-CCD camera during a two-step chronoamperometry measurement: 2 s at OCP and 8 s at 1.2 V *vs* Ag/AgCl. Magnification, X100; objective numerical aperture, 0.8; gain, 1; sensitivity, 350. The profiles were extracted from 90x4 pixel rectangles centred on the beads and, eventually, processed by subtracting the background signal as the average emission intensity of the initial 10 pixel. The background emission arises from homogeneous ECL. Data are averaged over a minimum of six beads (n ≥ 6).

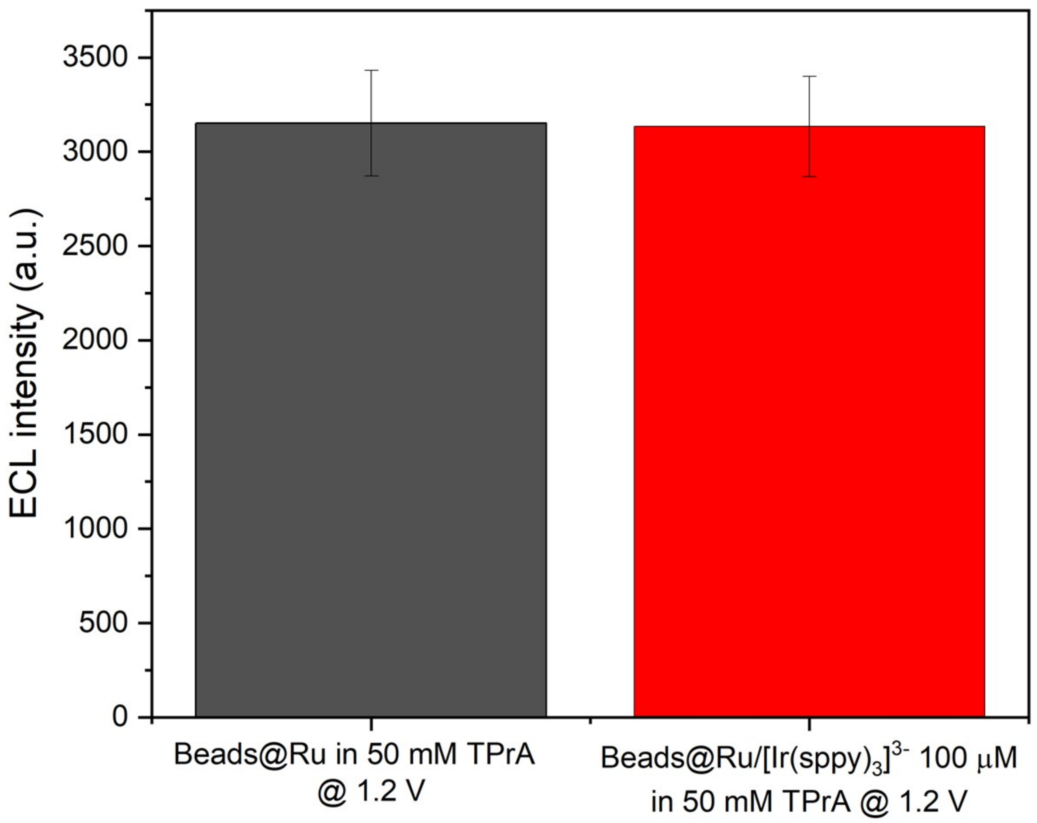

Figure S16. Comparison between ECL intensities of Ru@Beads (grey bar) and of Ru@Beads/$[Ir(sppy)_3]^{3-}$ at 100 µM concentration of $[Ir(sppy)_3]^{3-}$ (red bar) in a 0.3 M PB solution at pH 6.8 with 50 mM TPrA. The ECL intensities were obtained from ECL images captured using an EM-CCD camera during a two-step chronoamperometry measurement: 2 s at OCP and 8 s at 1.2 V *vs* Ag/AgCl. Magnification, X100; objective numerical aperture, 0.8; gain, 1; sensitivity, 350. Data are averaged over a minimum of six beads (n ≥ 6). Each bar represents the maximum value of the respective ECL profile and the error bars show the standard errors.

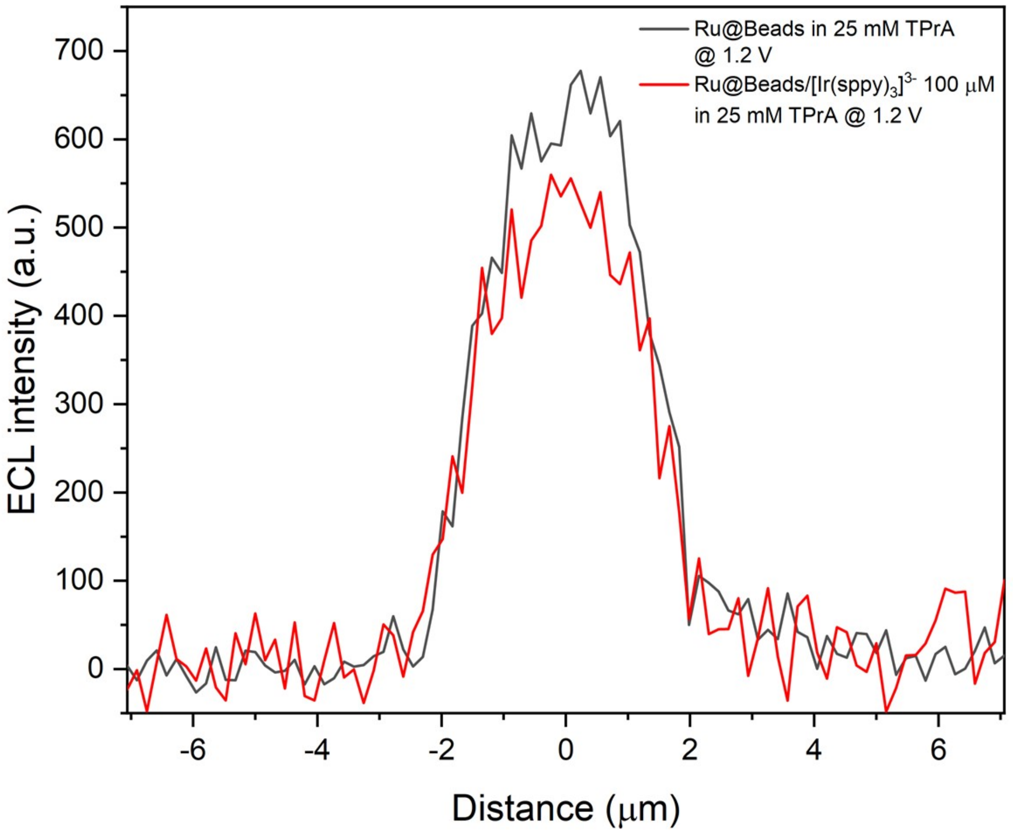

Figure S17. Comparison between ECL profiles of Ru@Beads (grey line) and of Ru@Beads/$[Ir(sppy)_3]^{3-}$ at 100 μM concentration of $[Ir(sppy)_3]^{3-}$ (red line) in a 0.3 M PB solution at pH 6.8 with 25 mM TPrA. The ECL profiles were obtained from ECL images captured using an EM-CCD camera during a two-step chronoamperometry measurement: 2 s at OCP and 8 s at 1.2 V *vs* Ag/AgCl. Magnification, X100; objective numerical aperture, 0.8; gain, 1; sensitivity, 350. The profiles were extracted from 90x4 pixel rectangles centred on the beads and, eventually, processed by subtracting the background signal as the average emission intensity of the initial 10 pixel. The background emission arises from homogeneous ECL. Data are averaged over a minimum of six beads (n ≥ 6).

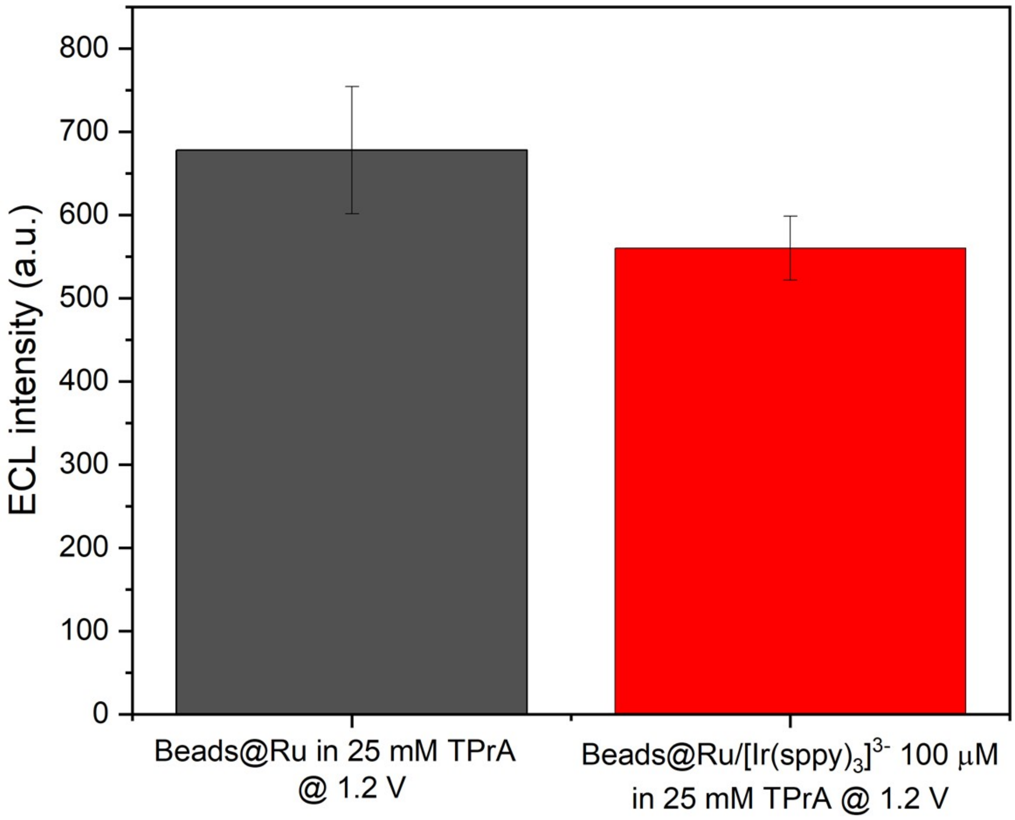

Figure S18. Comparison between ECL intensities of Ru@Beads (grey bar) and of Ru@Beads/$[Ir(sppy)_3]^{3-}$ at 100 µM concentration of $[Ir(sppy)_3]^{3-}$ (red bar) in a 0.3 M PB solution at pH 6.8 with 25 mM TPrA. The ECL intensities were obtained from ECL images captured using an EM-CCD camera during a two-step chronoamperometry measurement: 2 s at OCP and 8 s at 1.2 V *vs* Ag/AgCl. Magnification, X100; objective numerical aperture, 0.8; gain, 1; sensitivity, 350. Data are averaged over a minimum of six beads ($n \geq 6$). Each bar represents the maximum value of the respective ECL profile and the error bars show the standard errors.

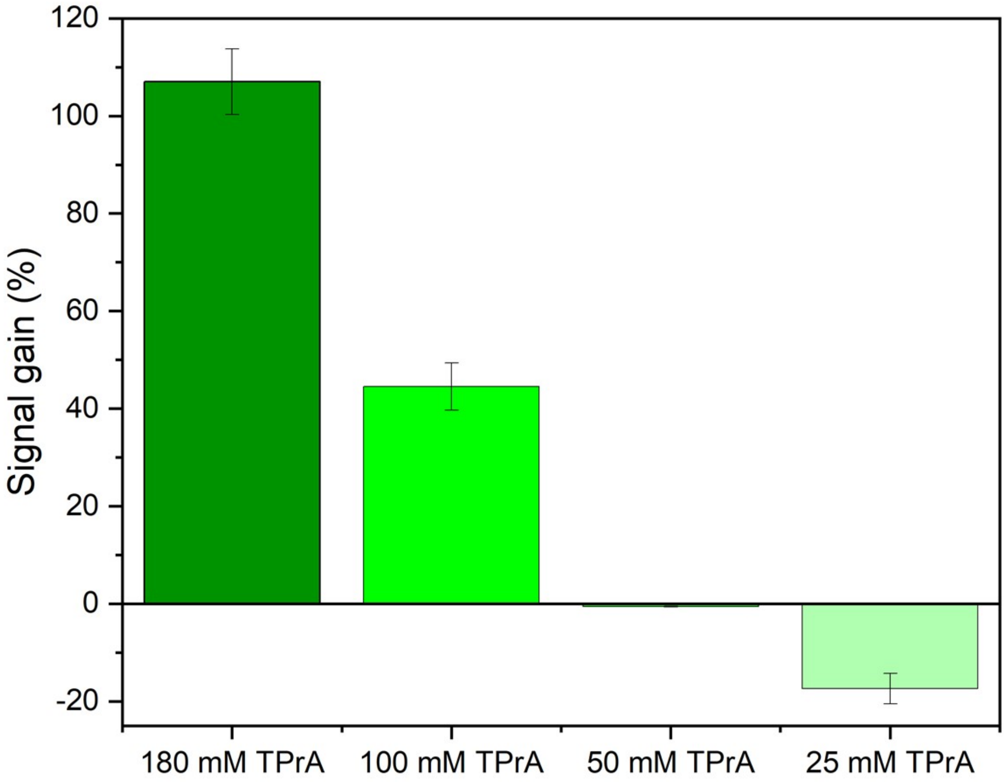

Figure S19. Comparison between ECL signal gain of Ru@Beads/$[Ir(sppy)_3]^{3-}$ with 100 µM $[Ir(sppy)_3]^{3-}$ compared to Ru@Beads, both in a 0.3 M PB solution at pH 6.8 with different TPrA concentrations: 180 mM (dark green bar), 100 mM (bright green bar), 50 mM (green bar), and 25 mM (mint green bar). Each bar represents the percentage difference between the maximum values of the ECL profiles of Ru@Beads/$[Ir(sppy)_3]^{3-}$ and Ru@Beads under the same working conditions. The ECL intensities of both, Ru@Beads and Ru@Beads/$[Ir(sppy)_3]^{3-}$, were obtained from ECL images captured using an EM-CCD camera during a two-step chronoamperometry measurement: 2 s at OCP and 8 s at 1.2 V vs Ag/AgCl. Magnification, X100; objective numerical aperture, 0.8; gain, 1; sensitivity, 255 when working in 180 mM TPrA; sensitivity, 350 for all the remaining TPrA concentrations. Data are averaged over a minimum of six beads ($n \geq 6$). The error bars show the standard errors.

9 Ru@Beads/[Ir(dfppy)$_2$(pt-TEG)]$^+$ ECL intensity at 1.5 V *vs* Ag/AgCl

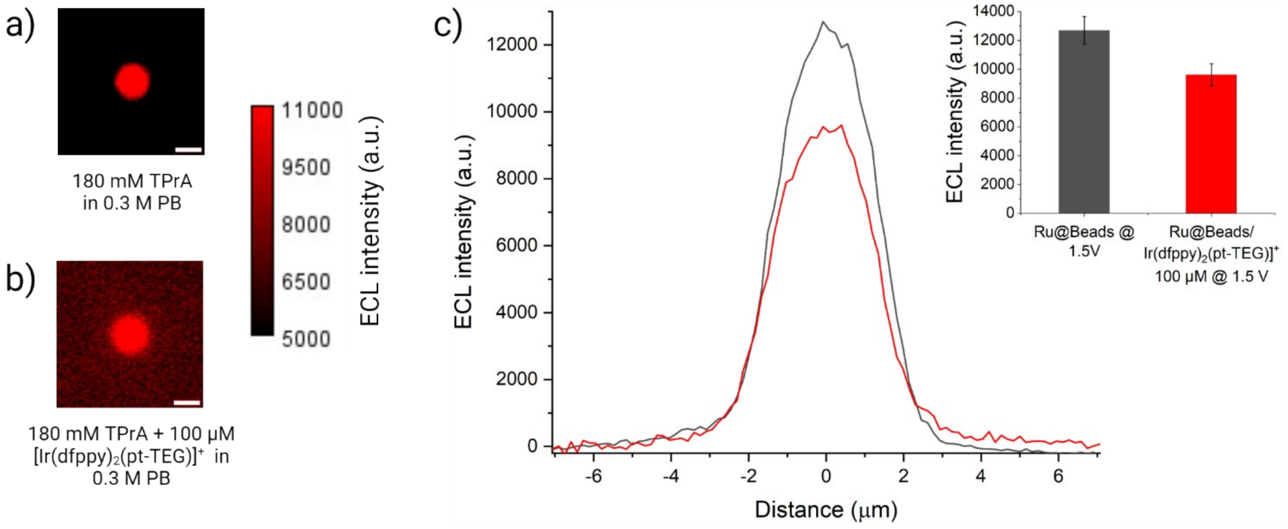

Figure S20. ECL images of a single 2.8 µm bead covalently labelled with $[Ru(bpy)_3]^{2+}$ in a 0.3 M PB solution at pH 6.8 with 180 mM TPrA (a) without (Ru@Beads) and (b) with 100 µM of $[Ir(dfppy)_2(pt\text{-}TEG)]^+$ (Ru@Beads/$[Ir(dfppy)_2(pt\text{-}TEG)]^+$). The background signal in (b) is generated by $[Ir(dfppy)_2(pt\text{-}TEG)]^{+*}$ following the conventional homogeneous ECL pathways. The images were obtained with an EM-CCD camera by recording the ECL signal for 10 s during a two-step chronoamperometry measurement: 2 s at open circuit potential (OCP) and 8 s at 1.5 V vs Ag/AgCl. Data are averaged over a minimum of six beads (n ≥ 6). Magnification, X100; objective numerical aperture, 0.8; gain, 1; sensitivity, 255; contrast scale: 5000 to 11000; scale bar: 3 µm. (c) Comparison between the single-bead ECL intensity profiles of Ru@Beads (grey line) and Ru@Beads/$[Ir(dfppy)_2(pt\text{-}TEG)]^+$ (red line). Inset: histogram of the comparison between the respective averaged maximum values of ECL intensity where the error bars show the standard error.

10 Ru@Beads/$[Ir(bt)_2(pt\text{-}TEG)]^+$ ECL intensity at 1.3 V *vs* Ag/AgCl

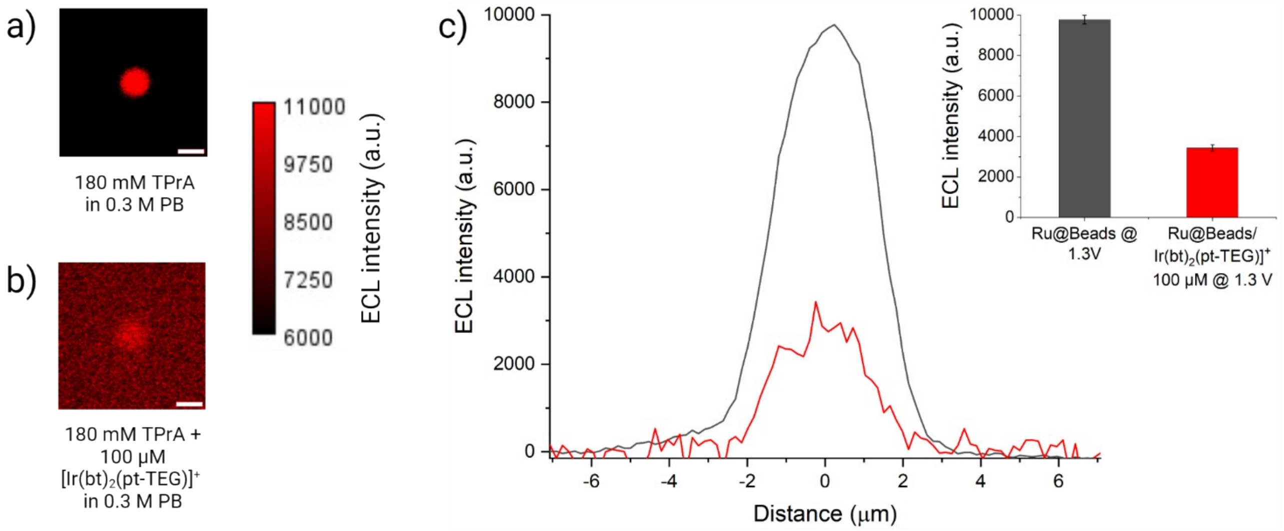

Figure S21. ECL images of a 2.8 µm single-bead covalently labelled with $[Ru(bpy)_3]^{2+}$ in a 0.3 M PB solution at pH 6.8 with 180 mM TPrA (a) without (Ru@Beads) and (b) with 100 µM of $[Ir(bt)_2(pt\text{-}TEG)]^+$. The background signal in (b) is generated by $[Ir(bt)_2(pt\text{-}TEG)]^{+*}$ following the conventional homogeneous ECL pathways. The images were obtained with an EM-CCD camera by recording the ECL signal for 10 s during a two-step chronoamperometry measurement: 2 s at OCP and 8 s at 1.3 V *vs* Ag/AgCl. Data are averaged over a minimum of six beads (n ≥ 6). Magnification, X100; objective numerical aperture, 0.8; gain, 1; sensitivity, 255; contrast scale, 6000 to 11000; scale bar, 3 µm. (c) Comparison between the single-bead ECL intensity profiles of Ru@Beads (grey line) and Ru@Beads/$[Ir(bt)_2(pt\text{-}TEG)]^+$ (red line). Inset: histogram of the comparison between the respective averaged maximum values of ECL emission where the error bars show the standard error.

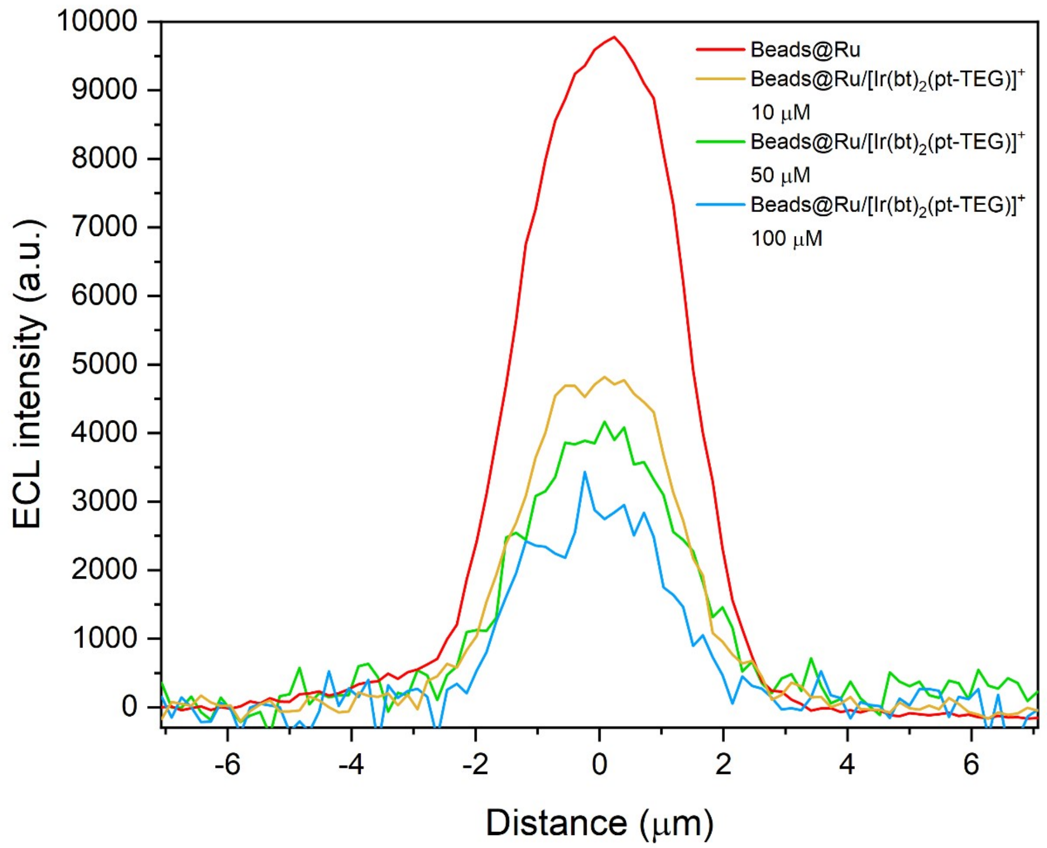

Figure S22. Comparison between ECL profiles of Ru@Beads (red line) and of Ru@Beads/$[Ir(bt)_2(pt\text{-}TEG)]^+$ in a 0.3 M PB solution at pH 6.8 with 180 mM TPrA and different $[Ir(bt)_2(pt\text{-}TEG)]^+$ concentrations: 10 µM (yellow line), 50 µM (green line) and 100 µM (blue line). The ECL profiles were obtained from ECL images captured using an EM-CCD camera during a two-step chronoamperometry measurement: 2 s at OCP and 8 s at 1.3 V *vs* Ag/AgCl. Magnification, X100; objective numerical aperture, 0.8; gain, 1; sensitivity, 255. The profiles were extracted from 90x4 pixel rectangles centred on the beads and, eventually, elaborated by subtracting the background signal which is generated from homogeneous ECL. It is determined as the average value of the signal intensity over the first 10 pixels for Ru@Beads/$[Ir(bt)_2(pt\text{-}TEG)]^+$ at 10 and 50 µM concentration of metal complex while it is considered as the ECL profile of Beads/$[Ir(bt)_2(pt\text{-}TEG)]^+$ at 100 µM concentration. Data are averaged over a minimum of six beads (n ≥ 6).

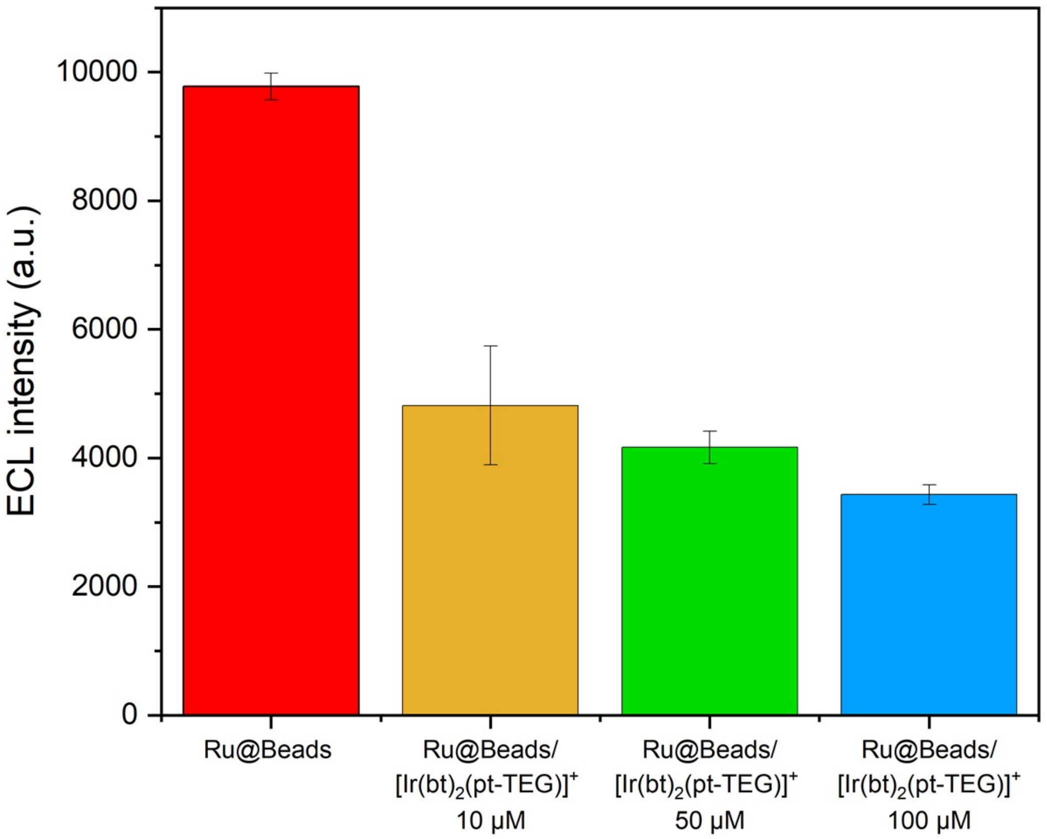

Figure S23. Comparison between ECL intensities of Ru@Beads (red bar) and of Ru@Beads/$[Ir(bt)_2(pt\text{-}TEG)]^+$ in a 0.3 M PB solution at pH 6.8 with 180 mM TPrA and different $[Ir(bt)_2(pt\text{-}TEG)]^+$ concentrations: 10 µM (yellow bar), 50 µM (green bar) and 100 µM (blue bar). The ECL intensities were obtained from ECL images captured using an EM-CCD camera during a two-step chronoamperometry measurement: 2 s at OCP and 8 s at 1.3 V *vs* Ag/AgCl. Magnification, X100; objective numerical aperture, 0.8; gain, 1; sensitivity, 255. Data are averaged over a minimum of six beads (n ≥ 6). Each bar represents the maximum value of the respective ECL profile and the error bars show the standard errors.

11 Ru@Beads/$[Ir(bt)_2(pt\text{-}TEG)]^+$ ECL intensity at 1.2 V *vs* Ag/AgCl

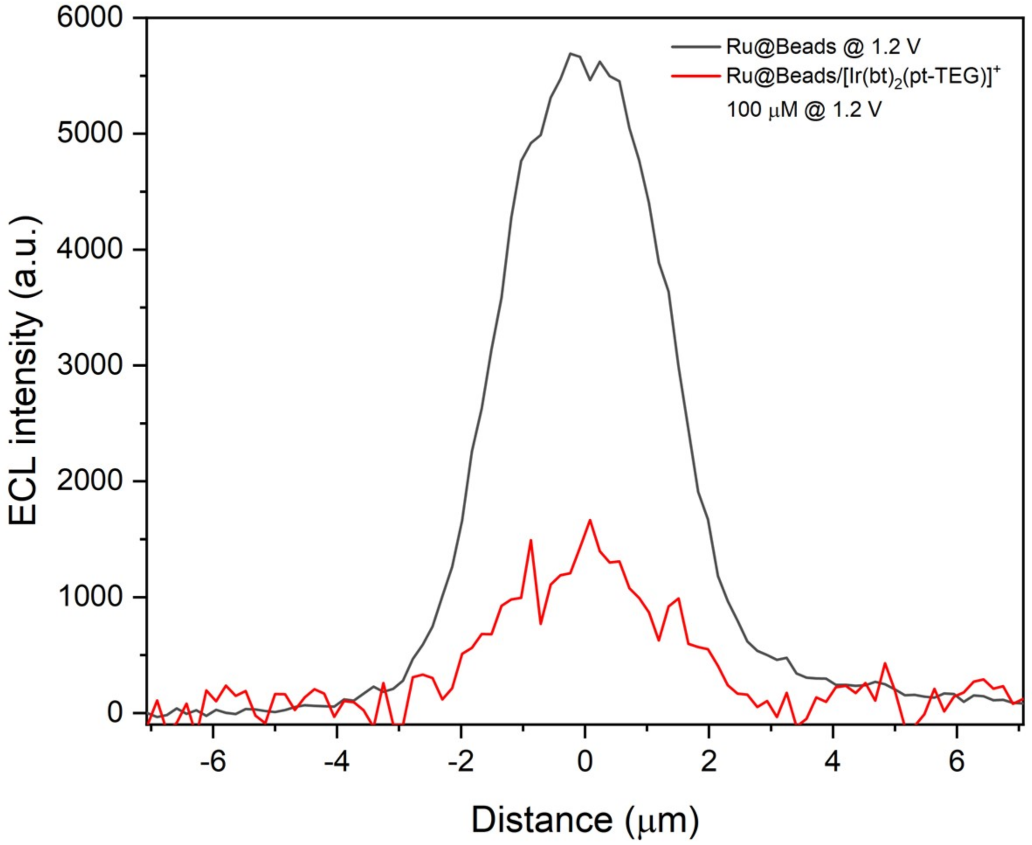

Figure S24. Comparison between ECL profiles of Ru@Beads (grey line) and of Ru@Beads/$[Ir(bt)_2(pt\text{-}TEG)]^+$ in a 0.3 M PB solution at pH 6.8 with 180 mM TPrA and 100 µM of $[Ir(bt)_2(pt\text{-}TEG)]^+$ (red line). The ECL profiles were obtained from ECL images captured using an EM-CCD camera during a two-step chronoamperometry measurement: 2 s at OCP and 8 s at 1.2 V *vs* Ag/AgCl. Magnification, X100; objective numerical aperture, 0.8; gain, 1; sensitivity, 255. The profiles were extracted from 90x4 pixel rectangles centred on the beads and, eventually, elaborated by subtracting the background signal which is generated from homogeneous ECL and that is considered as the ECL profile of Beads/$[Ir(bt)_2(pt\text{-}TEG)]^+$. Data are averaged over a minimum of six beads ($n \geq 6$).

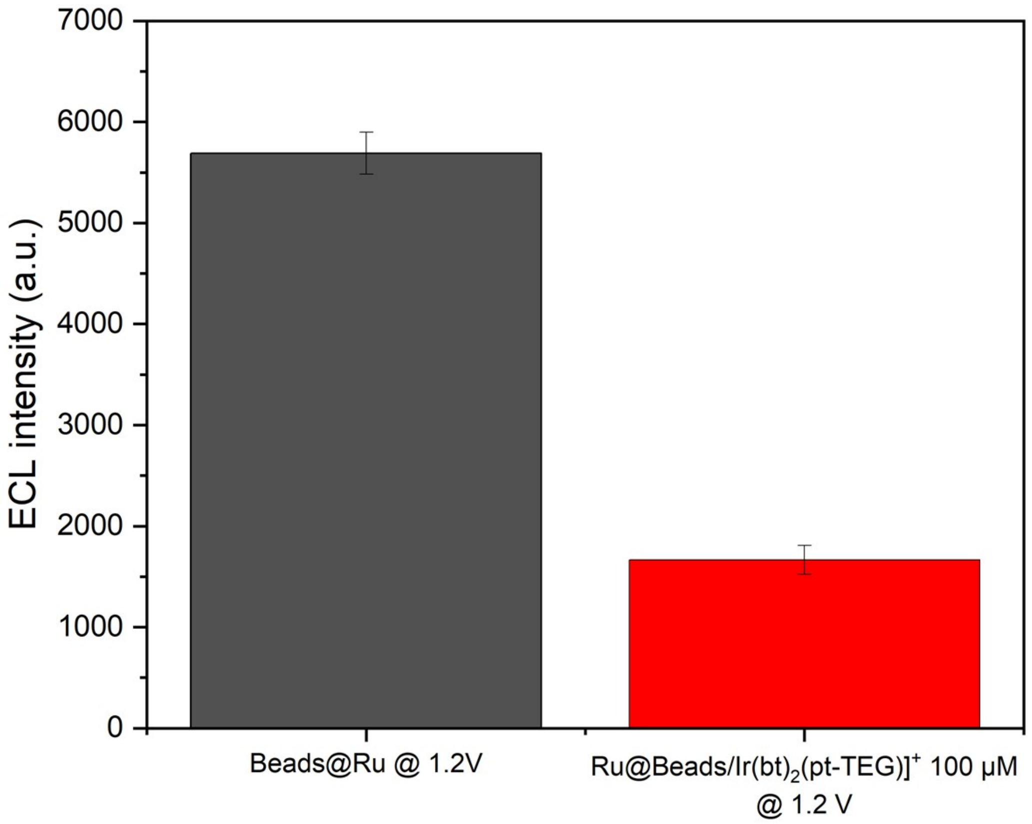

Figure S25. Comparison between ECL intensities of Ru@Beads (grey bar) and of Ru@Beads/$[Ir(bt)_2(pt\text{-}TEG)]^+$ in a 0.3 M PB solution at pH 6.8 with 180 mM TPrA and 100 μM of $[Ir(bt)_2(pt\text{-}TEG)]^+$ (red bar). The ECL intensities were obtained from ECL images captured using an EM-CCD camera during a two-step chronoamperometry measurement: 2 s at OCP and 8 s at 1.2 V *vs* Ag/AgCl. Magnification, X100; objective numerical aperture, 0.8; gain, 1; sensitivity, 255. Data are averaged over a minimum of six beads ($n \geq 6$). Each bar represents the maximum value of the respective ECL profile and the error bars show the standard errors.

12 Ru@Beads/$[Fe(bpy)_3]^{2+}$ ECL intensity at 1.2 V *vs* Ag/AgCl

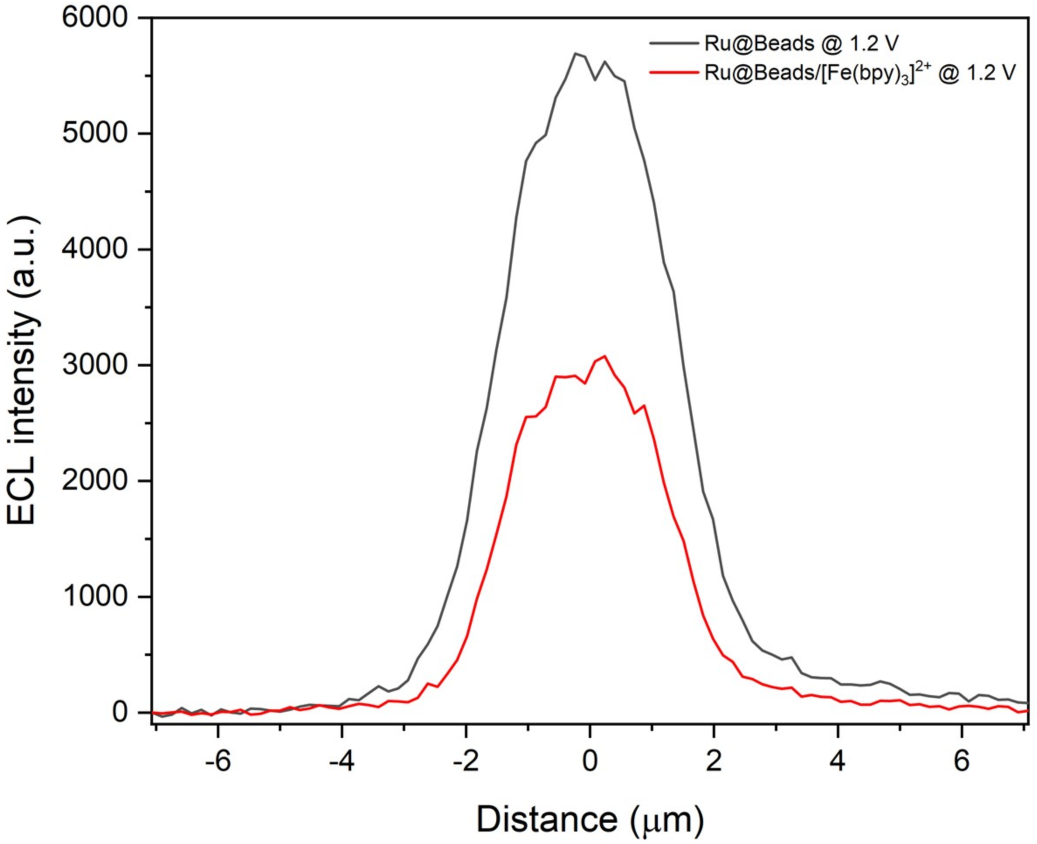

Figure S26. Comparison between ECL profiles of Ru@Beads (grey line) and of Ru@Beads/$[Fe(bpy)_3]^{2+}$ in a 0.3 M PB solution at pH 6.8 with 180 mM TPrA and 100 µM of $[Fe(bpy)_3]^{2+}$ (red line). The ECL profiles were obtained from ECL images captured using an EM-CCD camera during a two-step chronoamperometry measurement: 2 s at OCP and 8 s at 1.2 V *vs* Ag/AgCl. Magnification, X100; objective numerical aperture, 0.8; gain, 1; sensitivity, 255. The profiles were extracted from 90x4 pixel rectangles centred on the beads and, eventually, elaborated by subtracting the background signal which is generated from homogeneous ECL and that is determined as the average value of the signal intensity over the initial 10 pixels. Data are averaged over a minimum of six beads (n ≥ 6).

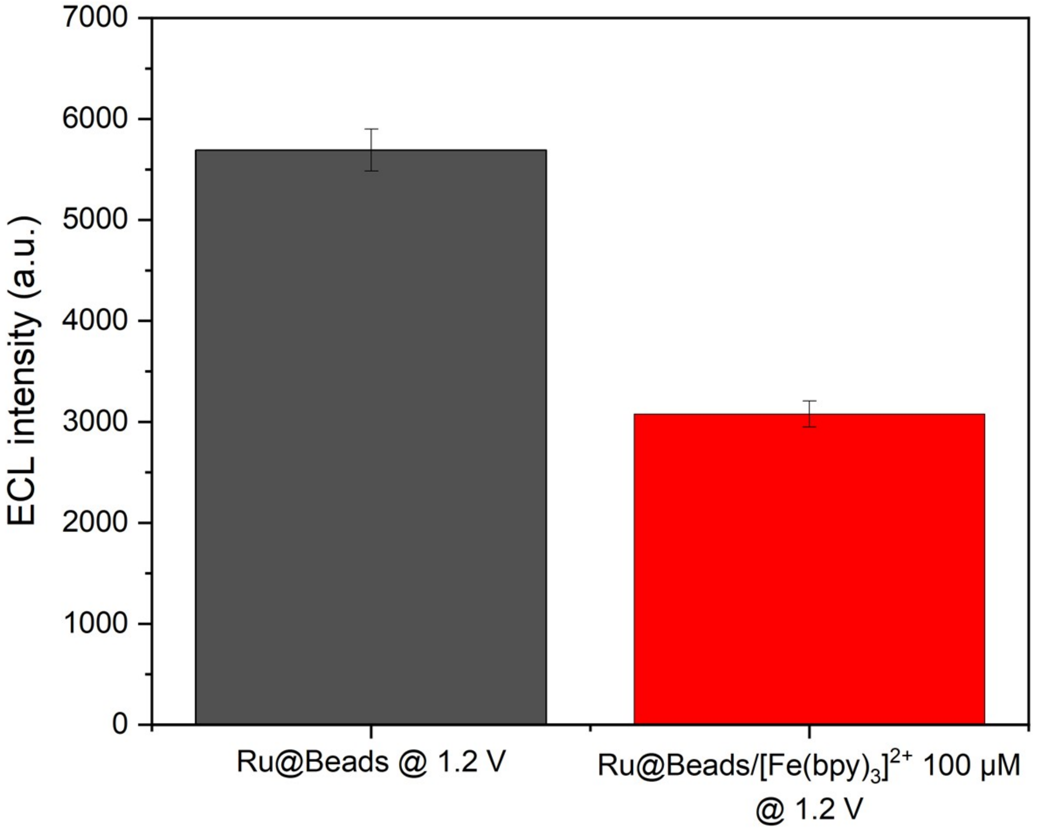

Figure S27. Comparison between ECL intensities of Ru@Beads (grey bar) and of Ru@Beads/$[Fe(bpy)_3]^{2+}$ in a 0.3 M PB solution at pH 6.8 with 180 mM TPrA and 100 µM of $[Fe(bpy)_3]^{2+}$ (red bar). The ECL intensities were obtained from ECL images captured using an EM-CCD camera during a two-step chronoamperometry measurement: 2 s at OCP and 8 s at 1.2 V *vs* Ag/AgCl. Magnification, X100; objective numerical aperture, 0.8; gain, 1; sensitivity, 255. Data are averaged over a minimum of six beads (n ≥ 6). Each bar represents the maximum value of the respective ECL profile and the error bars show the standard errors.

13 Ir-mediated bead-based simulated ECL

13.1 General modelling strategy

In order to provide a rationale to account for the observed ECL enhancement and quenching phenomena, a numerical model is proposed. Owing to the large number of species and reactions involved, presented in Section 3 of the Supplementary Information, we propose here a simplified model. It combines a steady-state formalism to describe the evolution of the Ru species at the bead surface with a COMSOL simulation of the reactions involved during the electrochemical oxidation of species in solution (i.e., Ir- or Fe-based redox mediator and the TPrA co-reactant).

In brief, owing to the swift diffusion of solution species and their fast reaction with Ru labels anchored at the bead surface, it is considered that the reactions on the beads surface between the freely diffusing Ir- or Fe-based redox mediator and the surface-anchored Ru labels do not significantly impact the concentration of the redox mediator in solution. Hence, the contributions to the beads ECL signal are expected to be dictated by steady-state approximation of the reactive Ru intermediates, namely Ru^{2+}, Ru^{+} and Ru^{2+*}. These contributions to the ECL emission result from the reaction of these surface bound labels with relevant solution species. As will be shown below, depending on the different oxidation and reduction standard potentials of the Ir or Fe complexes, presented in Fig. 2, different mathematical expression of the ECL emission are employed, relying solely on the concentration of the species already present or generated in solution.

The next step consists in evaluating the spatio-temporal concentration profiles of the various species present or generated in solution (i.e., TPrA and Ir or Fe complexes). This is achieved by a COMSOL 1D simulation of a cyclic voltammetry or chronoamperometric experiment. From the simulated concentration profiles, it is then possible, using the steady-state expression of the Ru species, to evaluate the ECL emission.

13.2 Steady-state expression of the Ru ECL emission

For the sake of simplicity, we will denote the Ir species initially present in solution as Ir(III), while its oxidised form will be denoted Ir(IV). Depending on the E^{o} of this redox couple Ir(IV)/Ir(III) compared to the electrode potential, we would end up dealing, in the vicinity of the anodically polarized electrode surface, with a solution containing Ir(III) or a mixture of Ir(III) and Ir(IV). As the solution also contains TPrA, which can be oxidised at the electrode surface, the composition of the solution near the electrode surface would be influenced by the reduction standard potential of the Ir species in relation to the oxidation potential of $TPrA^{\bullet}$. Consequently, we may have a solution that also includes Ir(II) species close to the electrode surface, as seen in the case of the two $[Ir(C^\wedge N)_2(pt\text{-}TEG)]^{+}$ complexes.

Note: 'Ir(II)' is used here as a convenient abbreviation for the reduced $[Ir(C^\wedge N)_2(pt\text{-}TEG)]^{0}$ species, but the LUMO of the $[Ir(C^\wedge N)_2(pt\text{-}TEG)]^{+}$ complexes is predominantly ligand centred,[1] so the reduced form should be considered $[Ir^{III}(C^\wedge N)_2(pt\text{-}TEG)]^{0}$, with the additional negative change distributed over one or more of the ligands.

These limiting cases can be encountered depending on the redox mediator E^{o}:

(a) In the case of $[Ir(sppy)_3]^{3-}$, the Ir(II) species cannot be formed by the oxidation of the $TPrA^{\bullet}$ by Ir(III), and only Ir(III) and Ir(IV) species are involved.
(b) In the case of the two $[Ir(C^\wedge N)_2(pt\text{-}TEG)]^{+}$ complexes when applying a potential lower than that needed to oxidise Ir(III), the latter species can still homogeneously oxidize $TPrA^{\bullet}$; hence only Ir(III) and Ir(II) species are involved.
(c) In the case of the $[Fe(bpy)_3]^{2+}$, all redox states can be considered: Fe(II), Fe(III) and the reduced Fe(I) species.

Assuming that the reaction rates with Ru labels are controlled by mass transfer of the species in solution to the bead surface, the equations for reactive species of Ru are then expressed depending on the situation (a), (b) or (c).

13.2.1 Case of $[Ir(sppy)_3]^{3-}$

In this case, as the Ir(III) is not oxidizing enough to oxidize the $TPrA^{\bullet}$ radical, the solution phase contains, apart from the TPrA reactive species, only Ir(III) and Ir(IV) in solution. The possible reactions involving the Ru species are the following ones:

$$[Ru(bpy)_3]^{2+} + TPrA^{\bullet} \rightarrow [Ru(bpy)_3]^{+} + Im^{+} \quad (1.3)$$

$$[Ru(bpy)_3]^{+} + TPrA^{\bullet +} \rightarrow [Ru(bpy)_3]^{2+*} + TPrA \quad (1.4)$$

$$[Ru(bpy)_3]^{2+*} \rightarrow [Ru(bpy)_3]^{2+} + hv\ (620\ nm) \quad (1.5)$$

$$[Ru(bpy)_3]^{+} + [Ir(sppy)_3]^{2-} \rightarrow [Ru(bpy)_3]^{2+*} + [Ir(sppy)_3]^{3-} \quad (2.8)$$

While the reactions occurring at the electrode surface and in solution for Ir and TPrA are:

$$TPrA \rightarrow TPrA^{\bullet +} + e^{-} \quad (2.1)$$

$$[Ir(sppy)_3]^{3-} \rightarrow [Ir(sppy)_3]^{2-} + e^{-} \quad (2.2)$$

$$[Ir(sppy)_3]^{3-} + TPrA^{\bullet +} \rightleftharpoons [Ir(sppy)_3]^{2-} + TPrA \quad (2.3)$$

$$TPrA^{\bullet +} \rightarrow TPrA^{\bullet} + H^{+} \quad (2.4)$$

$$[Ir(sppy)_3]^{2-} + TPrA^{\bullet} \rightarrow [Ir(sppy)_3]^{3-*} + Im^{+} \quad (2.5)$$

$$[Ir(sppy)_3]^{3-*} \rightarrow [Ir(sppy)_3]^{3-} + hv\ (515\ nm) \quad (2.6)$$

$$TPrA^{\bullet} \rightarrow Im^{+} + e^{-} \quad (2.7)$$

From the reactions proceeding at the bead surface, the surface concentration of Ru species can be expressed:

$$\frac{d[Ru(bpy)_3{}^{+}]}{dt} = -k_{1.4}[TPrA^{\bullet +}][Ru(bpy)_3{}^{+}] + k_{1.3}[TPrA^{\bullet}][Ru(bpy)_3{}^{2+}] - k_{2.8}[Ir(IV)][Ru(bpy)_3{}^{+}]$$

$$\frac{d[Ru(bpy)_3{}^{2+}]}{dt} = -k_{1.3}[TPrA^{\bullet}][Ru(bpy)_3{}^{2+}] + k_{1.5}[Ru(bpy)_3{}^{2+*}]$$

$$\frac{d[Ru(bpy)_3{}^{2+*}]}{dt} = k_{1.4}[TPrA^{\bullet +}][Ru(bpy)_3{}^{+}] + k_{2.8}[Ir(IV)][Ru(bpy)_3{}^{+}] - k_{1.5}[Ru(bpy)_3{}^{2+*}]$$

Where k_i corresponds to the kinetic rate constant of the reaction number (i).
Upon steady-state approximation (dRu/dt=0) they transform into:

$$k_{1.3}[TPrA^{\bullet}][Ru(bpy)_3{}^{2+}] = k_{1.5}[Ru(bpy)_3{}^{2+*}]$$

$$[Ru(bpy)_3{}^{2+}] = \frac{k_{1.5}[Ru(bpy)_3{}^{2+*}]}{k_{1.3}[TPrA^{\bullet}]}$$

$$[Ru(bpy)_3{}^{+}] = \frac{k_{1.5}[Ru(bpy)_3{}^{2+*}]}{k_{1.4}[TPrA^{\bullet +}] + k_{2.8}[Ir(IV)]}$$

and the ECL emission rate is given by:

$$ECL \propto \frac{dh\nu}{dt} = k_{1.5}[Ru(bpy)_3{}^{2+*}]$$

The mass conservation of Ru species (considering that the Ru^{2+*} concentration is negligible owing to its fast emission rate) yields:

$$[Ru(bpy)_3]_{total} = \Gamma^0 = [Ru(bpy)_3{}^{+}] + [Ru(bpy)_3{}^{2+}] = \frac{k_{1.5}[Ru(bpy)_3{}^{2+*}]}{k_{1.4}[TPrA^{\bullet +}] + k_{2.8}[Ir(IV)]} + \frac{k_{1.5}[Ru(bpy)_3{}^{2+*}]}{k_{1.3}[TPrA^{\bullet}]}$$

Generating:

$$ECL \propto \frac{dh\nu}{dt} = \frac{\Gamma^0}{\frac{1}{k_{1.4}[TPrA^{\bullet +}] + k_{2.8}[Ir(IV)]} + \frac{1}{k_{1.3}[TPrA^{\bullet}]}}$$

(13.1)

Noteworthy, in the absence of Ir species, the highest ECL intensity is achieved by maximising both $TPrA^{\bullet}$ and $TPrA^{\bullet+}$ species, as demonstrated in earlier bead-based ECL imaging systems.[4]
The ECL emission can be evaluated from the knowledge of the spatio-temporal evolution of the concentration of [$TPrA^{\bullet+}$], [Ir(IV)] and [$TPrA^{\bullet}$], which are evaluated by COMSOL as discussed in the next Section 13.3.

13.2.2 Case of $[Ir(C\text{^}N)_2(pt\text{-}TEG)]^+$

In this case, the Ir(III) is oxidizing enough to oxidize the $TPrA^{\bullet}$ radical. On the other hand, at 0.9 V or 1.2 V the Ir(III) species are assumed not to be converted into Ir(IV) species. Then the solution phase contains, apart from the TPrA reactive species, only the Ir(III) with Ir(II).
The possible reactions involving the Ru species at the bead surface are the following:

$$[Ru(bpy)_3]^{2+} + TPrA^{\bullet} \rightarrow [Ru(bpy)_3]^{+} + Im^{+} \quad (1.3)$$

$$[Ru(bpy)_3]^{+} + TPrA^{\bullet +} \rightarrow [Ru(bpy)_3]^{2+*} + TPrA \quad (1.4)$$

$$[Ru(bpy)_3]^{2+*} \rightarrow [Ru(bpy)_3]^{2+} + h\nu\ (620\ nm) \quad (1.5)$$

$$[Ru(bpy)_3]^{2+} + [Ir(C\text{^}N)_2(pt\text{-}TEG)] \rightarrow [Ru(bpy)_3]^{+} + [Ir(C\text{^}N)_2(pt\text{-}TEG)]^{+} \quad (3.11)$$

While the reactions considered at the electrode or in solution for the TPrA and Ir species are the following:

$$TPrA \rightarrow TPrA^{\bullet +} + e^{-} \quad (3.1)$$

$$[Ir(C\text{^}N)_2(pt\text{-}TEG)]^{+} \rightarrow [Ir(C\text{^}N)_2(pt\text{-}TEG)]^{2+} + e^{-} \quad (3.2)$$

$$TPrA^{\bullet +} \rightarrow TPrA^{\bullet} + H^{+} \quad (3.4)$$

$$[Ir(C\text{^}N)_2(pt\text{-}TEG)]^{+} + TPrA^{\bullet} \rightarrow [Ir(C\text{^}N)_2(pt\text{-}TEG)] + Im^{+} \quad (3.5)$$

$$[Ir(C\text{^}N)_2(pt\text{-}TEG)] + TPrA^{\bullet +} \rightarrow [Ir(C\text{^}N)_2(pt\text{-}TEG)]^{+} + TPrA \quad (3.6)$$

$$\left[Ir(C^\wedge N)_2(pt\text{-}TEG)\right]^{2+} + TPrA^{\bullet} \rightarrow \left[Ir(C^\wedge N)_2(pt\text{-}TEG)\right]^{+*} + Im^{+} \quad (3.7)$$

$$\left[Ir(C^\wedge N)_2(pt\text{-}TEG)\right]^{+*} \rightarrow \left[Ir(C^\wedge N)_2(pt\text{-}TEG)\right]^{+} + hv \quad (3.8)$$

$$\left[Ru(bpy)_3\right]^{2+} + TPrA^{\bullet} \rightarrow \left[Ru(bpy)_3\right]^{+} + Im^{+} \quad (3.9)$$

From the reactions involving the surface concentration of Ru species, it follows:

$$\frac{d\left[Ru(bpy)_3{}^{+}\right]}{dt} = -k_{1.4}[TPrA^{\bullet+}][Ru(bpy)_3{}^{+}] + k_{1.3}[TPrA^{\bullet}][Ru(bpy)_3{}^{2+}] + k_{3.11}[Ir(II)][Ru(bpy)_3{}^{2+}]$$

$$\frac{d\left[Ru(bpy)_3{}^{2+}\right]}{dt} = -k_{1.3}[TPrA^{\bullet}][Ru(bpy)_3{}^{2+}] + k_{1.5}[Ru(bpy)_3{}^{2+*}] - k_{3.11}[Ir(II)][Ru(bpy)_3{}^{2+}]$$

$$\frac{d\left[Ru(bpy)_3{}^{2+*}\right]}{dt} = k_{1.4}[TPrA^{\bullet+}][Ru(bpy)_3{}^{+}] - k_{1.5}[Ru(bpy)_3{}^{2+*}]$$

Upon steady-state approximation they transform into:

$$k_{1.4}[TPrA^{\bullet+}][Ru(bpy)_3{}^{+}] = k_{1.5}[Ru(bpy)_3{}^{2+*}]$$

$$[Ru(bpy)_3{}^{+}] = \frac{k_{1.5}[Ru(bpy)_3{}^{2+*}]}{k_{1.4}[TPrA^{\bullet+}]}$$

$$[Ru(bpy)_3{}^{2+}] = \frac{k_{1.5}[Ru(bpy)_3{}^{2+*}]}{k_{1.3}[TPrA^{\bullet}] + k_{3.11}[Ir(II)]}$$

and the ECL emission rate is given by:

$$ECL \propto \frac{dhv}{dt} = \frac{\Gamma^0}{\dfrac{1}{k_{1.4}[TPrA^{\bullet+}]} + \dfrac{1}{k_{1.3}[TPrA^{\bullet}] + k_{3.11}[Ir(II)]}}$$
(13.2)

13.2.3 Case of $[Fe(bpy)_3]^{2+}$

Noteworthy, for the Ru@Beads/$[Fe(bpy)_3]^{2+}$ system all three redox states of $[Fe(bpy)_3]^{2+}$ are accessible (which are abbreviated here as Fe(I), Fe(II) and Fe(III); see note above). A combination of Equations 13.1 and 13.2 yields:

$$ECL \propto \frac{dhv}{dt} = \frac{\Gamma^0}{\dfrac{1}{k_{1.4}[TPrA^{\bullet+}] + k_{2.8}[Fe(III)]} + \dfrac{1}{k_{1.3}[TPrA^{\bullet}] + k_{3.11}[Fe(I)]}}$$
(13.3)

13.3 COMSOL simulation of the solution species concentration profiles

In this step, we conducted simulations of the 1D concentration profiles of the solution species produced during the electrochemical measurement, namely chronoamperometry or cyclic voltammetry, in a solution containing initially both TPrA and Ir(III) species. We carried out the simulations considering the complete set

of solution-phase and electrode reactions outlined in Section 3 (Reaction Schemes, see above) where the reaction rate constants are provided in Table S2. These concentration profiles have been computed using COMSOL and the spatial distribution of the most relevant species (i.e., $TPrA^{•}$, $TPrA^{•+}$ and Ir(IV), Ir(III) or Ir(II)) are shown in Fig. S28a and S28b. These profiles are then exploited in Equations 13.1 and 13.2 to compute the ECL emission profiles presented in Fig. S28a and S28b which demonstrate the reproducibility of ECL boosting or scavenging behaviour, respectively, based on the rate constant provided in Table S2. Finally, for the $[Fe(bpy)_3]^{2+}$ case, the COMSOL simulation yields the $TPrA^{•}$, $TPrA^{•+}$, Fe(III), Fe(II) and Fe(I) concentration profiles that are exploited to compute the ECL intensity of Ru@Beads/$[Fe(bpy)_3]^{2+}$ via Equation 13.3.

Case of $[Ir(sppy)_3]^{3-}$

a

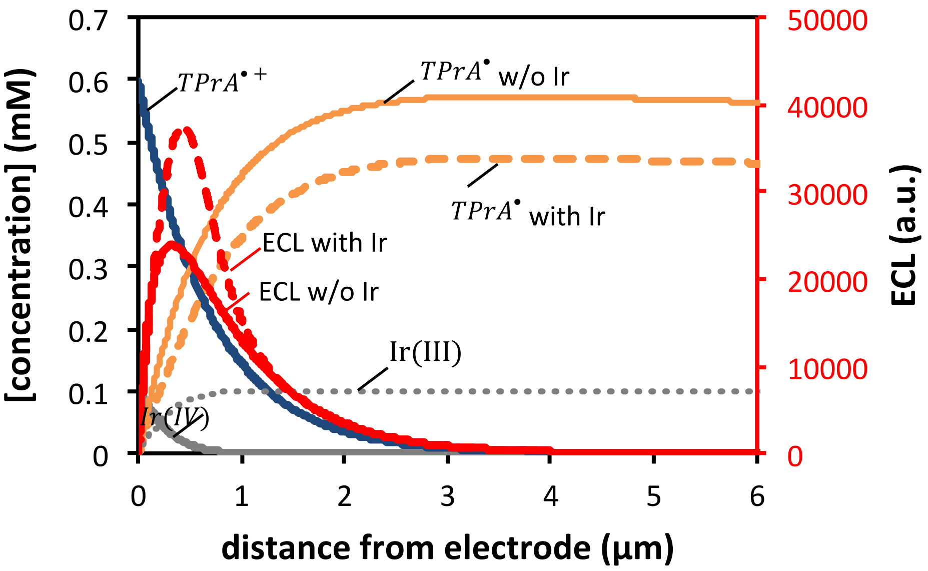

Case of $[Ir(C\^{}N)_2(pt\text{-}TEG)]^{+}$

b

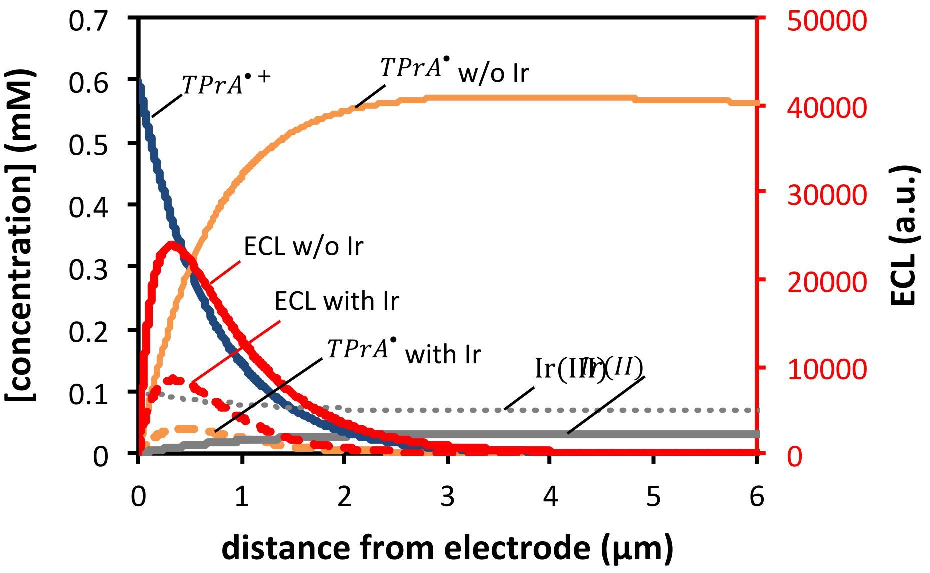

Case of [Fe(bpy)$_3$]$^{2+}$

c

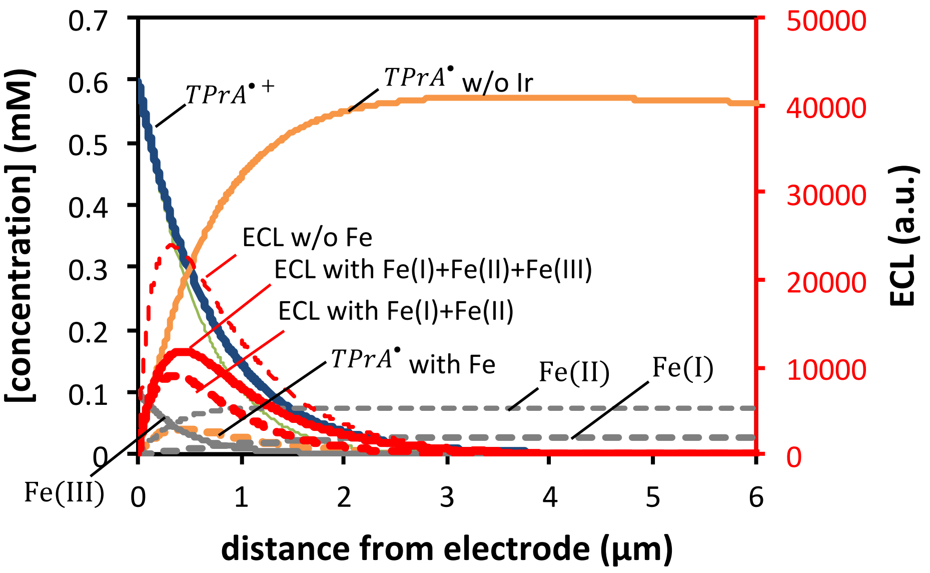

Fig. S28. The concentration profiles of various solution species, namely TPrA$^{\bullet+}$, TPrA$^{\bullet}$, Ir(III) and either (a) Ir(IV) or (b) Ir(II), with respect to the direction normal to the electrode surface were computed using COMSOL. The profiles simulate the concentration of the species generated at 1.2 V *vs* Ag/AgCl in a solution of 180 mM TPrA and in presence or absence

of 100 µM Ir(III) species. Using Equations S13.1 or S13.2, the ECL emission profiles are computed in the absence (plain red line) or presence (dashed red line) of Ir(III) species. The values of the rate constants employed for the simulations and for Equations 13.1 and 13.2 are listed in the Table S2 below. (c) Similar ECL profiles computed using Equation 13.3 in presence of all three redox states of the Fe-based mediator (bold plain red line) and by neglecting the presence of the oxidising Fe(III) (bold dashed red line).

Fig. 28a shows that the introduction of a homogeneous redox mediator, such as an Ir(III) complex that can be converted in Ir(IV) upon electrooxidation, results in a slight scavenging of $TPrA^{\bullet}$ and yields an ECL enhancement when $k_{2.8} > k_{1.3}$. Notably, a slight decrease in ECL signal is observed if the mediated Ir(IV)/Ru(I) electron transfer is not considered. In Fig. 28b, the generation of Ir(II) species is shown to significantly scavenge $TPrA^{\bullet}$. This confines the $TPrA^{\bullet}$ radical in the vicinity of the electrode surface, inhibiting its propagation into the solution bulk. Interestingly, in the latter scenario, the Ir mediator is instrumental in impeding the diffusion of radical reactive species to the bulk, thereby preventing further radical chemistry processes. Eventually, this behaviour results in a sharp decrease of the ECL signal. In this context, the higher the Ru(II)/Ir(II) electron transfer $k_{3.11}$ rate constant (compared to $k_{1.3}$) and the less the ECL is quenched. In the case of $[Fe(bpy)_3]^{2+}$ being used as redox mediator, the plain ECL curve in Fig. 28c shows that involving all three redox states of the Fe-based redox mediator, (i.e., Fe(I), Fe(II) and Fe(III)) and using the same exchange rates as in Fig. 28a and 28b, the quenching is less pronounced than in absence of Fe(III) (bold dashed red line).

Reactions	Constant	Value
Reactions at the bead surface (steady-state assumption through Equations 13.1 and 13.2)		
$[Ru(bpy)_3]^{2+} + TPrA^{\bullet} \rightarrow [Ru(bpy)_3]^{+} + Im^{+}$	$k_{1.3}$	$3\cdot10^{8}$ $M^{-1}s^{-1}$
$[Ru(bpy)_3]^{+} + TPrA^{\bullet+} \rightarrow [Ru(bpy)_3]^{2+*} + TPrA$	$k_{1.4}$	10^{8} $M^{-1}s^{-1}$
$[Ru(bpy)_3]^{+} + [Ir(IV)] \rightarrow [Ru(bpy)_3]^{2+*} + [Ir(III)]$	$k_{2.8}$	$3\cdot10^{9}$ $M^{-1}s^{-1}$
$[Ru(bpy)_3]^{2+} + [Ir(II)] \rightarrow [Ru(bpy)_3]^{+} + [Ir(III)]$	$k_{3.11}$	$<10^{8}$ $M^{-1}s^{-1}$ for Fig. S28b; quenching is observed up to $7\cdot10^{9}$ $M^{-1}s^{-1}$
Reactions for solution species:<br>- involving Ir(IV) species		
$TPrA \rightarrow TPrA^{\bullet+} + e^{-}$	E°, ks	0.83 V, 0.01 cm s^{-1}
$TPrA^{\bullet+} \rightarrow TPrA^{\bullet} + H^{+}$	k_d	$2\cdot10^{3}$ s^{-1}
$TPrA^{\bullet} \rightarrow Im^{+} + e^{-}$	E^{0}_{rad}, ks_{rad}	-1.7 V, 1 cm s^{-1}
$[Ir(III)] \rightarrow [Ir(IV)] + e^{-}$	E^{0}_{IrOx}, ks_{Ir}	0.83 V, 0.1 cm s^{-1}
$[Ir(III)] + TPrA^{\bullet+} \rightleftarrows [Ir(IV)] + TPrA$	k_{fcat}, k_{bcat}	Not considered for $[Ir(C^N)_2(pt\text{-}TEG)]^{+}$. 10^{4} (k_{fcat}), 10^{9} (k_{bcat}) $M^{-1}s^{-1}$ for $[Ir(sppy)_3]^{3-}$, $[Fe(bpy)_3]^{2+}$
$[Ir(IV)] + TPrA^{\bullet} \rightarrow [Ir(III)]^{*} + Im^{+}$	kq_{Irox}	10^{8} $M^{-1}s^{-1}$
$[Ir(III)]^{*} \rightarrow [Ir(IV)] + h\nu_{Ir}$	$k_{em,Ir}$	10^{8} s^{-1}

-involving Ir(II) species		
$[Ir(II)] \rightarrow [Ir(III)] + e^-$	E^o_{IrRed}, ks_{Ir}	Not considered for $[Ir(sppy)_3]^{3-}$ or -1.7 V; 1 cm s^{-1} for $[Ir(C\hat{}N)_2(pt\text{-}TEG)]^+$, $[Fe(bpy)_3]^{2+}$
$[Ir(II)] + TPrA^{\bullet +} \rightarrow [Ir(III)]^* + TPrA$		
$[Ir(III)] + TPrA^{\bullet} \rightarrow [Ir(II)] + Im^+$	kq_{Ir}	0 or 10^8 M^{-1} s^{-1}
$[Ir(II)] + [Ir(IV)] \rightarrow [Ir(III)]^* + [Ir(III)]$	$k_{ann,Ir}$	10^8 M^{-1} s^{-1}

Table S2. Reactions and their characteristic constants used to simulate by a 1D COMSOL model the concentration profiles of the solution species and, from Equations 12.1 and 12.2, the ECL signal.

14 Collective beads experiment

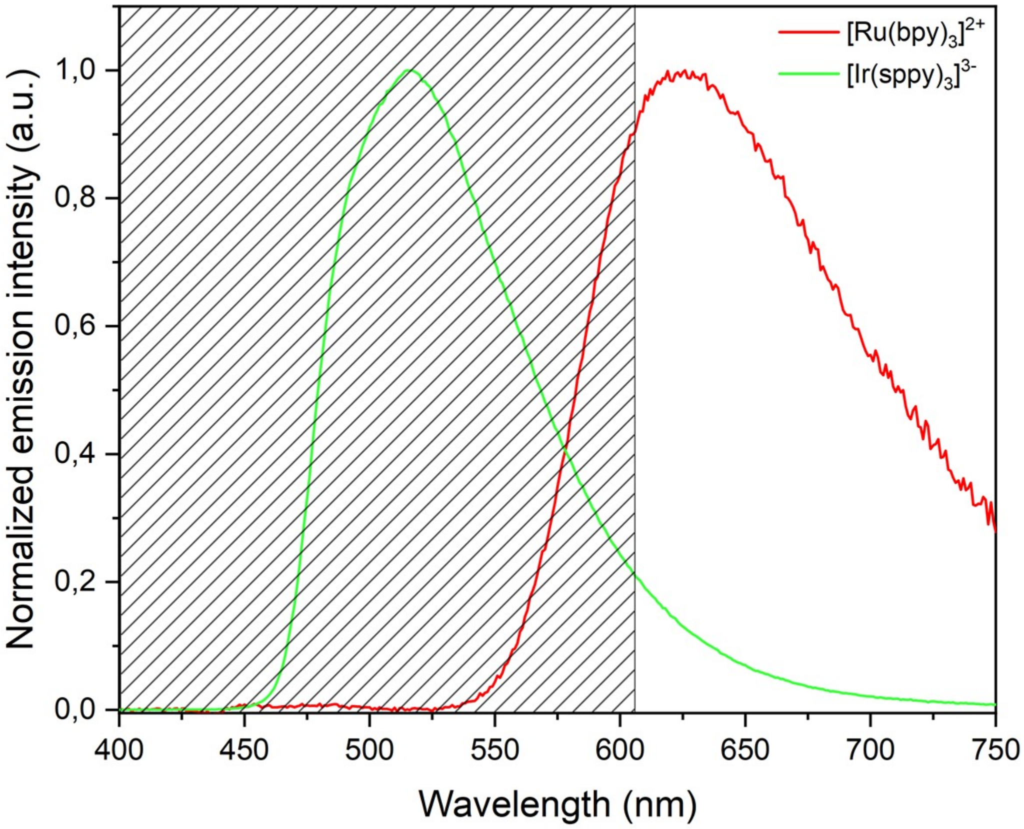

Figure S29. Normalized emission spectra of $[Ir(sppy)_3]^{3-}$ (green line) and $[Ru(bpy)_3]^{2+}$ (red line), respectively. The utilized optical filter cuts off all the emission wavelengths below 606 nm (dark region), preventing most of the $[Ir(sppy)_3]^{3-}$ homogeneous ECL to strike the PMT while the peak of the $[Ru(bpy)_3]^{2+}$ emission spectrum is preserved.

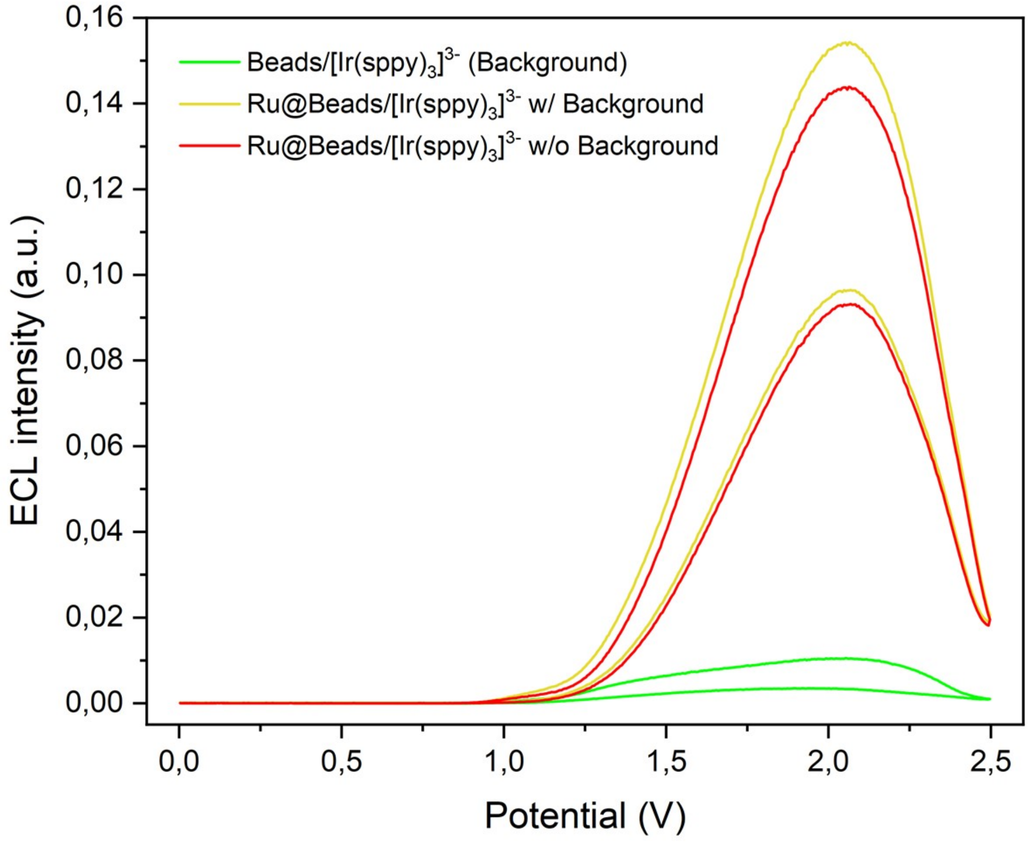

Figure S30. CV-ECL measurements performed on Beads/[Ir(sppy)$_3$]$^{3-}$ (i.e., background signal; green line) and on Ru@Beads/[Ir(sppy)$_3$]$^{3-}$ at 50 µM concentration of [Ir(sppy)$_3$]$^{3-}$ (including background, yellow line; background subtracted, red line), both in a 0.3 M PB solution at pH 6.8 with 180 mM TPrA. The ECL intensity values of Ru@Beads/[Ir(sppy)$_3$]$^{3-}$ for each data point along the x-axis were corrected by subtracting the ECL intensity values of Beads/[Ir(sppy)$_3$]$^{3-}$ at the corresponding potential values. The working electrode potential was scanned at 100 mV/s from OCP up to 2.5 V (*vs* Ag/AgCl), back to 0 V (*vs* Ag/AgCl) and, eventually, terminating the cycle at OCP. The optical filter (λ_{cut-on} = 606 nm) was employed for every measurement. Data are averaged over two different measurements.

15 References

1 L. Chen, D. J. Hayne, E. H. Doeven, J. Agugiaro, D. J. D. Wilson, L. C. Henderson, T. U. Connell, Y. H. Nai, R. Alexander, S. Carrara, C. F. Hogan, P. S. Donnelly and P. S. Francis, *Chem. Sci.*, 2019, **10**, 8654–8667.

2 E. Kerr, D. J. Hayne, L. C. Soulsby, J. C. Bawden, S. J. Blom, E. H. Doeven, L. C. Henderson, C. F. Hogan and P. S. Francis, *Chem. Sci.*, 2022, **13**, 469–477.

3 E. Kerr, S. Knezevic, P. S. Francis, C. F. Hogan, G. Valenti, F. Paolucci, F. Kanoufi and N. Sojic, *ACS Sensors*, 2023, **8**, 933–939.

4 T. Saji and S. Aoyagui, *Electroanal. Chem. Interfacial Electrochem.*, 1975, **60**, 1–10.

5 M. Ocafrain, M. Devaud, M. Troupel and J. Perichon, *Electrochim. Acta*, 1997, **42**, 99–105.
